# Long-term *in vivo* chimeric cells tracking in non-human primate

**DOI:** 10.1093/procel/pwad049

**Published:** 2023-09-27

**Authors:** Junmo Wu, Yu Kang, Xiang Luo, Shaoxing Dai, Yuxi Shi, Zhuoyao Li, Zengli Tang, Zhenzhen Chen, Ran Zhu, Pengpeng Yang, Zifan Li, Hong Wang, Xinglong Chen, Ziyi Zhao, Weizhi Ji, Yuyu Niu

**Affiliations:** State Key Laboratory of Primate Biomedical Research, Institute of Primate Translational Medicine, Kunming University of Science and Technology, Kunming 650500, China; Yunnan Key Laboratory of Primate Biomedical Research, Kunming 650500, China; State Key Laboratory of Primate Biomedical Research, Institute of Primate Translational Medicine, Kunming University of Science and Technology, Kunming 650500, China; Yunnan Key Laboratory of Primate Biomedical Research, Kunming 650500, China; State Key Laboratory of Primate Biomedical Research, Institute of Primate Translational Medicine, Kunming University of Science and Technology, Kunming 650500, China; Yunnan Key Laboratory of Primate Biomedical Research, Kunming 650500, China; State Key Laboratory of Primate Biomedical Research, Institute of Primate Translational Medicine, Kunming University of Science and Technology, Kunming 650500, China; Yunnan Key Laboratory of Primate Biomedical Research, Kunming 650500, China; State Key Laboratory of Primate Biomedical Research, Institute of Primate Translational Medicine, Kunming University of Science and Technology, Kunming 650500, China; Yunnan Key Laboratory of Primate Biomedical Research, Kunming 650500, China; State Key Laboratory of Primate Biomedical Research, Institute of Primate Translational Medicine, Kunming University of Science and Technology, Kunming 650500, China; Yunnan Key Laboratory of Primate Biomedical Research, Kunming 650500, China; State Key Laboratory of Primate Biomedical Research, Institute of Primate Translational Medicine, Kunming University of Science and Technology, Kunming 650500, China; Yunnan Key Laboratory of Primate Biomedical Research, Kunming 650500, China; State Key Laboratory of Primate Biomedical Research, Institute of Primate Translational Medicine, Kunming University of Science and Technology, Kunming 650500, China; Yunnan Key Laboratory of Primate Biomedical Research, Kunming 650500, China; State Key Laboratory of Primate Biomedical Research, Institute of Primate Translational Medicine, Kunming University of Science and Technology, Kunming 650500, China; Yunnan Key Laboratory of Primate Biomedical Research, Kunming 650500, China; State Key Laboratory of Primate Biomedical Research, Institute of Primate Translational Medicine, Kunming University of Science and Technology, Kunming 650500, China; Yunnan Key Laboratory of Primate Biomedical Research, Kunming 650500, China; State Key Laboratory of Primate Biomedical Research, Institute of Primate Translational Medicine, Kunming University of Science and Technology, Kunming 650500, China; Yunnan Key Laboratory of Primate Biomedical Research, Kunming 650500, China; State Key Laboratory of Primate Biomedical Research, Institute of Primate Translational Medicine, Kunming University of Science and Technology, Kunming 650500, China; Yunnan Key Laboratory of Primate Biomedical Research, Kunming 650500, China; State Key Laboratory of Primate Biomedical Research, Institute of Primate Translational Medicine, Kunming University of Science and Technology, Kunming 650500, China; Yunnan Key Laboratory of Primate Biomedical Research, Kunming 650500, China; State Key Laboratory of Primate Biomedical Research, Institute of Primate Translational Medicine, Kunming University of Science and Technology, Kunming 650500, China; Yunnan Key Laboratory of Primate Biomedical Research, Kunming 650500, China; State Key Laboratory of Primate Biomedical Research, Institute of Primate Translational Medicine, Kunming University of Science and Technology, Kunming 650500, China; Yunnan Key Laboratory of Primate Biomedical Research, Kunming 650500, China; State Key Laboratory of Primate Biomedical Research, Institute of Primate Translational Medicine, Kunming University of Science and Technology, Kunming 650500, China; Yunnan Key Laboratory of Primate Biomedical Research, Kunming 650500, China; Faculty of Life Science and Technology, Kunming University of Science and Technology, Kunming 650500, China

**Keywords:** pluripotent stem cells, bioluminescence imaging, non-human primates

## Abstract

Non-human primates (NHPs) are increasingly used in preclinical trials to test the safety and efficacy of biotechnology therapies. Nonetheless, given the ethical issues and costs associated with this model, it would be highly advantageous to use NHP cellular models in clinical studies. However, developing and maintaining the naïve state of primate pluripotent stem cells (PSCs) remains difficult as does *in vivo* detection of PSCs, thus limiting biotechnology application in the cynomolgus monkey. Here, we report a chemically defined, xeno-free culture system for culturing and deriving monkey PSCs *in vitro*. The cells display global gene expression and genome-wide hypomethylation patterns distinct from monkey-primed cells. We also found expression of signaling pathways components that may increase the potential for chimera formation. Crucially for biomedical applications, we were also able to integrate bioluminescent reporter genes into monkey PSCs and track them in chimeric embryos *in vivo* and *in vitro*. The engineered cells retained embryonic and extra-embryonic developmental potential. Meanwhile, we generated a chimeric monkey carrying bioluminescent cells, which were able to track chimeric cells for more than 2 years in living animals. Our study could have broad utility in primate stem cell engineering and in utilizing chimeric monkey models for clinical studies.

## Introduction

Non-human primates (NHPs), such as cynomolgus monkeys, have a high degree of similarity to humans compared to other animal models. These similarities manifest at the genetic, physiological, socio-behavioral, and central nervous system levels, making NHPs uniquely suitable for research into stem cell therapy (Zhao et al. 2019). This potential has been shown in the context of transplanting human pluripotent stem cell (PSC)-islets for diabetes treatment in NHPs (Du et al. 2022), human PSC-derived cardiovascular progenitor cells for heart disease in NHPs (Zhu et al. 2018), and autologous transplant in parkinsonian monkeys (Tao et al. 2021). NHP stem cell culture is potentially a critical model for the assessment of potential therapeutics in preclinical trials.

Conventional generation of stem cells from human and mouse blastocysts produces a developmentally advanced, or primed, stage of pluripotency. “Naïve” state PSCs resemble the preimplantation blastocyst inner cell mass (ICM) and have the potential to differentiate into primordial germ cells (Nichols and Smith 2009; Weinberger et al. 2016). “Primed” state PSCs resemble post-implantation epiblast (EPI) cells that have a low capacity to contribute to embryonic chimeras and a low competence for germline differentiation (Nichols and Smith 2009; Zhang et al. 2018). A “naïve-like” phenotype indicates that cultured cells have naïve properties or primed properties as previously defined (Weinberger et al. 2016). NHP stem cell research applications would be greatly facilitated by a system that robustly leads to a naïve-like and naïve state.

Recent studies (Yang et al., 2017a, b) showed that extended pluripotent stem cells (EPSCs) can generate both embryonic and extra-embryonic lineages in chimeras *in vivo*. The studies have also demonstrated that human EPSCs can generate functional human lineages, such as hepatocytes, via directed differentiation (Wang et al. 2020). Moreover, mouse EPSCs can generate blastocyst-like structures *in vitro* (Li et al. 2019), providing a valuable platform for exploring early embryogenesis while removing much of the ethical and logistical burdens of animal husbandry for research. These studies have highlighted the promising applications and advantages of EPSCs in basic and translational research. To date, however, the translational application of EPS cell-based therapies to patients has not been reported. Consequently, preclinical research, especially in NHP, is still needed, significantly increasing the cost and complexity of both research and preclinical trials. Despite our previous efforts (Chen et al. 2015; Kang et al. 2018), primate stem cell cultures are several significant challenges to overcome before PSCs can be used for therapeutic research.

Recent improvements in generating pluripotent cell states are now well established in rodents. In addition, conventional primate PSCs, such as monkey ESCs and induced pluripotent stem cells (iPSCs), more closely resemble post-implantation mouse epiblast stem cells (EpiSCs) in terms of gene expression profiles, signaling pathways required for proliferation, and intolerance to single-cell passaging (Martin 1981; Thomson et al. 1998; Takahashi et al. 2007; Yu et al. 2007; Liu et al. 2008; Kinoshita et al. 2021; Yu et al. 2021b). Nevertheless, the pluripotency of NHP PSCs remains a nascent field that cannot, as yet, supportsrobust preclinical trials. While the naïve or totipotent states observed in human and rodent cells can be obtained with specific culture media in other non-primate models, the translation of these outcomes to primate cells is often uninformative and inadequate for clinical insight (Chen et al. 2021). The foundational challenge has been to identify feeder-free and xeno-free culture medium that can maintain stable pluripotency. Additionally, downstream application has further been limited by the lack of available *in vivo* tracking methods.

Bioluminescence imaging (BLI) is a robust and versatile technique that enables noninvasive and longitudinal monitoring of gene expression, cellular localization, and molecular processes in live animals (Mezzanotte et al. 2017; Liu et al. 2021). In BLI, the enzymatic activity of luciferase, either expressed independently or as part of a reporter protein catalyzes the oxidation of a chemical substrate, resulting in the emission of light. Over the last two decades, BLI has emerged as a standard technique in mammalian disease models, enabling the visualization of gene expression patterns and the tracking of cellular and viral distributions within the living system (Prescher and Contag 2010). Nonetheless, the application of BLI in large animal models remains relatively uncommon, primarily due to the limited penetration of traditional fluorescent signals through thick tissues, which poses challenges for detection using imaging instruments. As a result, researchers have made advancements by developing synthetic d-luciferin analogs that exhibit significantly enhanced bioluminescence in animal. Notably, CycLuc1 (Evans et al. 2014) and AkaLumine (Iwano et al. 2018) have emerged as promising options. The utilization of the AkaLuc–AkaLumine system enables real-time video recording of brain bioluminescence in freely moving mice and marmosets. However, despite the utilization of the AkaLuc–AkaLumine system for imaging in mice and marmosets, the performance of this system in large animal models has yet to be validated and quantified. The validation of the AkaLuc–AkaLumine system for large animal models is particularly crucial, as BLI is extensively employed in lineage tracing, disease progression, and cell therapy monitoring.

In this study, we have established chemically defined and xeno-free culture system for generating cynomolgus monkey (monkey for short) PSCs. These xeno-free PSCs can be derived from blastocysts, converted from established PSC lines, or generated by somatic cell reprogramming. Moreover, these cells are capable of differentiating into all three germ layers as well as into reproductive germ-like cells, both *in vitro* and *in vivo*. Finally, by integrating AkaLuc, a bioengineered luciferase gene, into the genome of monkey stem cells, we successfully created a bioluminescent-labeled chimeric monkey model. This allowed us to track the proliferation and migration of chimeric cells within the monkey *in vivo*.

## Results

### Establishment of xeno-free culture conditions for monkey PSCs

We first sought to establish and maintain an efficient and convenient pluripotency protocol that meets the quality control requirements of stem cell research. Feeder-independent and defined culture systems improve the safety and efficiency of stem cell technology by reducing the number of variables in the system and the risk of cross-contamination (Ludwig and Thomson 2007). We found that xeno-free culture-primed PSCs initially attached and generated outgrowths but either differentiated or died before passaging. Feeder-free culture of monkey PSCs is thought to require alterations in the signaling pathways based on the existing culture systems. A culture condition containing Nodal was previously used to maintain iPSCs from marmosets (Vermilyea et al. 2017). Meanwhile, we aim to enhance the pluripotency of stem cells under xeno-free culture conditions. By referencing culture systems such as t2iL-Go (Guo et al. 2016), 5iLA (Theunissen et al. 2014), EPS (Yang et al., 2017b), and PXGL (Bredenkamp et al. 2019), we attempted to regulate the signaling pathways involved in the maintenance of monkey stem cells using different combinations (Fig. S1A). The Activin/Nodal signaling pathway and the Wnt pathway play crucial roles in the core transcriptional network of stem cells (Chen et al. 2008; Vallier et al. 2009). The *ESRRB* gene plays a critical regulatory role in maintaining pluripotency (Cornacchia et al. 2019) and during stem cell reprogramming (Adachi et al. 2018) and we use the expression of the *ESRRB* gene as a reference to screen xeno-free culture systems (Fig. S1B and S1C). Subsequently, we conducted a search for alternative matrix proteins to replace the inconsistent Matrigel, which contains unidentified animal components. We tried the effects of varying concentrations of Vitronectin XF on the long-term culture of stem cells. We found that only at high concentration of Vitronectin XF (50 ng/mL) culture, the cells could proliferate stably for a long time, which is a defined, xeno-free matrix that supports the growth of PSCs. Finally, we have discovered a new culture method suitable for monkey’s PSCs without a feeder cell layer. Specifically, we found that the use of Activin-A (10 ng/mL) and IWR-1 endo (2 μmol/L) are beneficial for monkey stem cell transformation in this culture medium, hereinafter referred to as xeno-free pluripotent stem cell (XF-PSC) medium. This feeder-independent and defined culture system enhances the safety and efficiency of stem cell technology, aligning with our initial goal of establishing an efficient and convenient monkey stem cell protocol.

**Figure 1.**
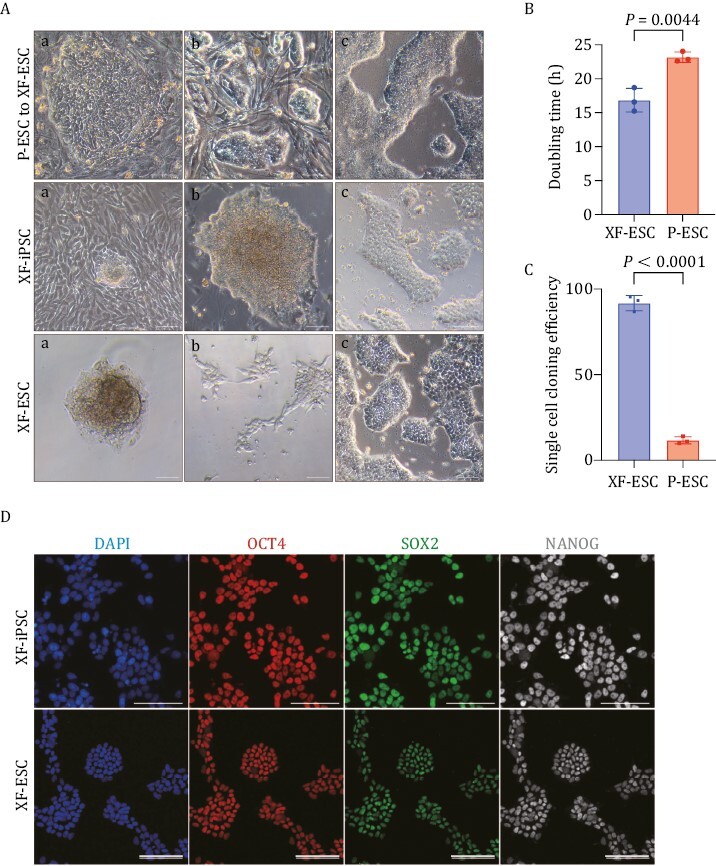
Establishment of a xeno-free culturing system for ESC lines and iPSC lines from monkey preimplantation embryos and fibroblasts. (A) Morphology of monkey ESCs colonies before and after conversion using the XF-PSC medium (upper) (a, cultured in a primed medium; b, conversion of cells; c, cultured in XF-PSC medium). Establishment of monkey iPSCs and ESCs from fibroblast (middle) (a, fibroblast reprogramming; b, iPSC clone; c, iPSCs cultured in XF-PSC medium) and monkey day 6 embryos (lower) (a, outgrowth; b, isolated single cell; c, ESCs cultured in XF-PSC medium). Scale bar, 100 μm. (B) Analysis of doubling time of primed ESCs and xeno-free ESCs. *n* = 3 biologically independent samples, data are mean with SD, unpaired two-tailed *t*-test, *P* = 0.0044. (C) Single-cell cloning efficiency of primed ESCs and xeno-free ESCs. *n* = 3 biologically independent samples, data are mean with SD, unpaired two-tailed *t*-test, *P* < 0.0001. (D) Immunostaining of pluripotency marker genes expression in establishment monkey iPSCs (upper). Immunostaining of pluripotency marker genes expression in establishment monkey ESCs (lower). Scale bar, 100 μm.

ESC, iPSC, and nuclear transfer embryonic stem cells (NTESC) exhibit similar lineage-specific gene expression and have great potential for disease modeling and drug therapy (Zhao et al. 2017).Therefore, we chose to initially establish iPSC, NTESC, and ESC lines for testing culture medium(Kang et al. 2018). When derived under xeno-free medium, primed iPSC (P-iPSC), primed nuclear transfer embryonic stem cell (P-NTESC), and primed embryonic stem cell (P-ESC), henceforth referred to as xeno-free induced pluripotent stem cell (XF-iPSC), XF-NTESC, and xeno-free embryonic stem cell (XF-ESC), exhibited rapid proliferation and stabilized within 4–6 passages (Figs. 1A and S1D). By this stage, they had formed compact colonies with smooth edges. The colonies maintained stable growth kinetics and routinely passaged 2–3 D (1:3 passaging ratio) after extended culture. We found that three XF-ESC lines showed faster growth than P-ESCs (Fig. 1B) and high single-cell cloning efficiency (*n* = 3) (Fig. 1C). Meanwhile, XF-NTESC, XF-iPSC, and XF-ESC have similar morphology (Figs. 1A and S1D) and expressed pluripotency genes marker (Fig. S1E).

Collectively, our results demonstrate that we established chemically defined and xeno-free culture conditions that support the maintenance of monkey PSCs of various genetic backgrounds.

### Establishment of XF-ESC lines and XF-iPSC lines from monkey preimplantation embryos and fibroblasts

As a control experiment, we next attempted to generate XF-iPSCs from monkey fibroblasts by reprogramming using our XF-PSC system. After 10–14 days, XF-iPSC-like colonies emerged that could be further propagated in the XF-PSC medium (Fig. 1A). We also confirmed the co-expression of the pluripotent markers OCT4, NANOG, and SOX2 in XF-iPSC colonies (Fig. 1D).

At the same time, we investigated deriving XF-ESC lines from monkey preimplantation embryos. From six early blastocysts [6 days post-fertilization (d.p.f.6)], three cell lines were established (Fig. 1A). XF-ESCs also expressed pluripotency genes (Fig. 1D). *In vivo*, XF-ESCs and XF-iPSCs formed mature teratomas that contained cell types of the three germ layers (Fig. S1F). *In vitro*, XF-ESC and XF-iPSCs differentiated into cells expressing genes representative of cell types of the three germ layers (Fig. S1G; Table S1). Furthermore, XF-ESCs and XF-iPSCs were maintained in culture long-term, while exhibiting normal karyotypes (Fig. S1H).

Collectively, XF-iPSCs derived from fibroblasts exhibited morphologically similarities to XF-ESCs derived from embryos. Both cell types retained the expression of pluripotent marker genes and showed differentiation potential. These results indicate that XF-ESCs and XF-iPSCs can be derived from preimplantation monkey embryos and somatic cells using our chemically defined and xeno-free culturing medium.

### Expansion of XF-ESCs with pluripotency features

**Figure 2.**
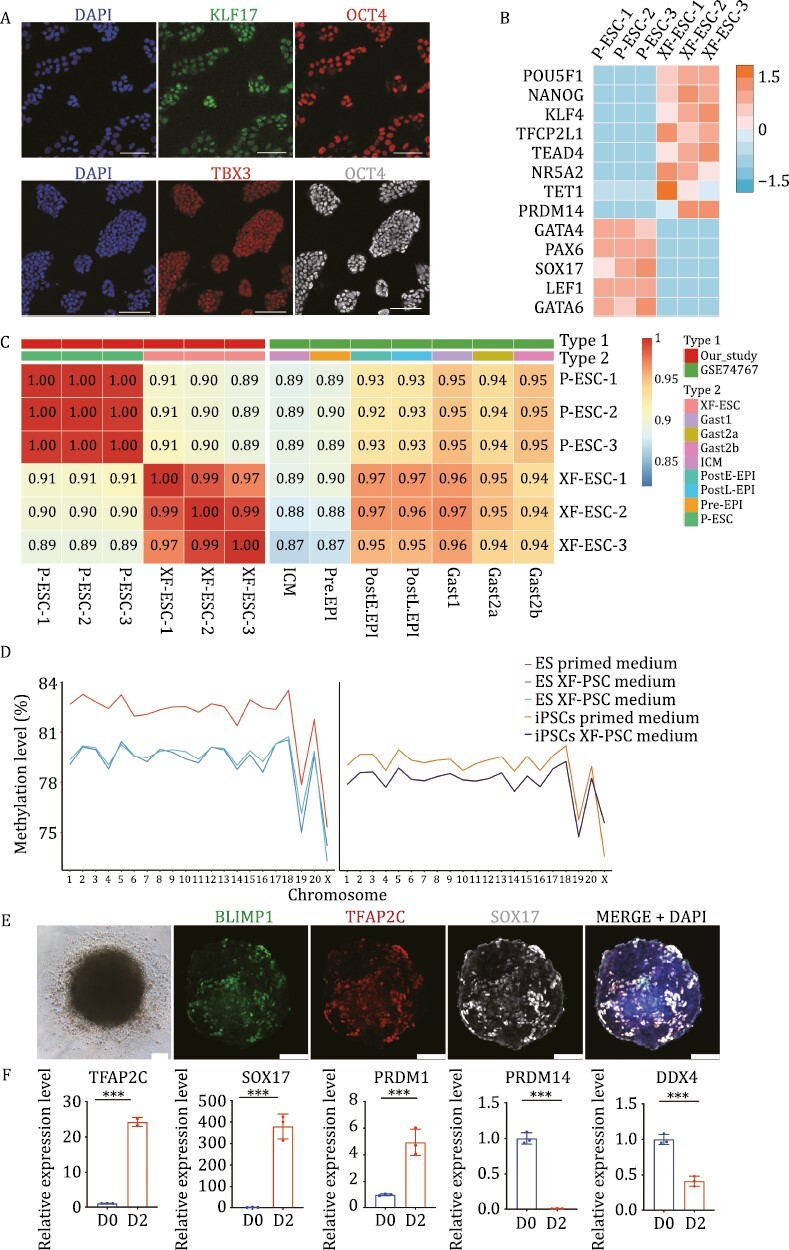
Expansion of pluripotency features from XF-ESCs. (A) IF staining of KLF17, TBX3, and OCT4 marker genes expression in monkey XF-ESCs after conversion using XF-PSC medium. Scale bar, 100 μm. (B) Heatmap of naïve state pluripotency-related genes expression of XF-ESCs and P-ESCs (Chen et al. 2015). (C) Heatmap of the correlation coefficients among XF-ESCs, P-ESCs, and reported by GSE74767. (D) The average methylation levels of each chromosome. (E) Bright-field (BF) images of PGCLCs and IF staining of day 8 PGCLCs. Similar results were obtained in three independent experiments (scale bar, 100 μm). (F) Gene expression dynamics during PGCLCs induction from XF-ESCs. *n* = 3, biological replicates, data are mean with SD, unpaired two-tailed *t*-test, ***P < 0.001.

We then tested whether cells cultured in our new chemically defined medium express naïve-like pluripotency features, such as expression of naïve stage genes. In the XF-PSC culture system, XF-ESCs expressed naïve pluripotency-related markers KLF17 and TBX3 (Figs. 2A and S2A). IF staining revealed the expression of KLF4 and DPPA3 in a subset of cells (Fig. S2B). Furthermore, real-time quantitative polymerase chain reaction (RT-qPCR) analysis indicated higher expression levels of naïve stage markers TFCP2L1, KLF17, and DPPA3 compared to the primed culture system (Fig. S2C). Conversely, the expression level of XIST, a marker of primed pluripotency, was found to be lower in the XF-PSC culture system than in the primed culture medium. Meanwhile, higher expression of KLF4, DNMT3A, and DNMT3B markers was detected in XF-ESCs transcriptome data (Fig. S2D). We then assessed the global gene expression profile of XF-ESCs, which revealed that they were distinct from P-ESCs (Fig. S2E and S2F). Expression of the naïve markers KLF4, TFCP2L1, TEAD4, NR5A2, TET1, and PRDM14 was increased and the lineage differentiation state markers GATA4, PAX6, SOX17, LEF1, and GATA6 was reduced in XF-ESCs (Fig. 2B). We also identified genes that were differentially expressed in xeno-free compared to P-ESCs (Fig. S2F). The function of these genes was largely related to several signaling pathways (e.g., phosphatidylinositol 3-kinase [PI3K]-Akt and mitogen-activated protein kinase [MAPK] signaling pathways) that were upregulated in XF-ESCs (Fig. S2G). In contrast, because of the addition of IWR-1, the Wnt signaling pathways were downregulated in XF-ESCs, which was consistent with previous findings. Meanwhile, specific metabolic pathways linked to the naïve state in mouse and human cells (e.g., glutathione metabolism) were newly expressed in XF-ESCs. To evaluate the relationship between XF-ESCs and the cells of monkey epiblast lineage, we examined the expression patterns of monkey EPI and monkey naïve state PSCs onto genic genes (Fang et al. 2014; Nakamura et al. 2016), which are able to delineate the stage along with the epiblast development (Figs. 2C and S2H). Compared with naïve state PSCs and primed-state PSCs, some of our XF-ESCs tended to resemble the expression profile of naïve state PSC and post-implantation early monkeys EPI cells. Furthermore, we transferred the established iPSC line and ESC line into the primed medium and found that compared with XF-iPSCs and XF-ESCs, the P-iPSCs and P-ESCs showed high levels of DNA methylation (Fig. 2D).

Next, we investigated whether primordial germ cell-like cells (PGCLCs) can be induced from XF-ESCs using chemical inhibition. In the early primitive streak stage of monkey embryos (d.p.f.13–d.p.f.17), the first cluster of monkey PGCs can be detected as SOX17+ and TFAP2C+ cells at the nascent primitive streak (Niu et al. 2019). To confirm PGCLC identity, we checked, and found, that BLIMP1, SOX17, and TFAP2C proteins were co-expressed using immunostaining in XF-ESC differentiation cultures (Fig. 2E). We also verified that a range of germ cell markers increased their expression and primed pluripotency gene expression decreased as measured by RT-qPCR (Fig. 2F). Collectively, these features constitute recognized hallmarks of monkey PGCLCs (Niu et al. 2019; Sakai et al. 2020).

Taken together, these observations suggest that XF-ESCs display enhanced pluripotency features. We previously modulated specific pathway activities (e.g., MAPK and PI3K-AKT) in donor XF-ESCs and suggested that this change might improve the efficiency of chimera formation (Tan et al. 2021). We found that P53 pathway activities in XF-ESCs may be induced by crowding hypersensitivity and mechanical cell competition (Wagstaff et al. 2016). Repression of gene expression may further improve efficiency of chimera formation (Nichols et al. 2022). Meanwhile, XF-ESCs have the ability to directly differentiate into PGCLCs.

### Chimeric contribution of XF-ESCs to peri- and post-implantation monkey embryos

While multiple luciferase-expressing animal models of human diseases have been reported and even made commercially available (Dothager et al. 2009; Mezzanotte et al. 2017), the application of bioluminescent imaging techniques for the *in vivo* imaging of chimeric NHP cells remains limited (Li et al. 2013; Mezzanotte et al. 2017; Momcilovic and Shackelford 2018). Therefore, we used a lentiviral vector consisting of a cytomegalovirus (CMV) promoter, which drives the expression of AkaLuc, a type of luciferase reporter gene (Fig. S3A). This is followed by the elongation factor-1 alpha (EF-1α) promoter, which regulates the expression of copGFP, a type of green fluorescent protein (GFP) (CMV-AkaLuc-EF-1α-copGFP). The configuration of this plasmid structure allows for simultaneous reporting of gene expression levels in XF-ESCs through luminescence and fluorescence, thus providing a means to fluorescently label these cells. CMV-AkaLuc-EF-1α-copGFP+ cells were continuously screened by flow cytometry during the process of cell proliferation. XF-ESCs were passaged over 60 times and were found to still maintain normal cell morphology (Fig. S3B). CMV-AkaLuc-EF-1α-copGFP+ cells were finally purified and still maintaining pluripotency (Fig. S3C).

**Figure 3.**
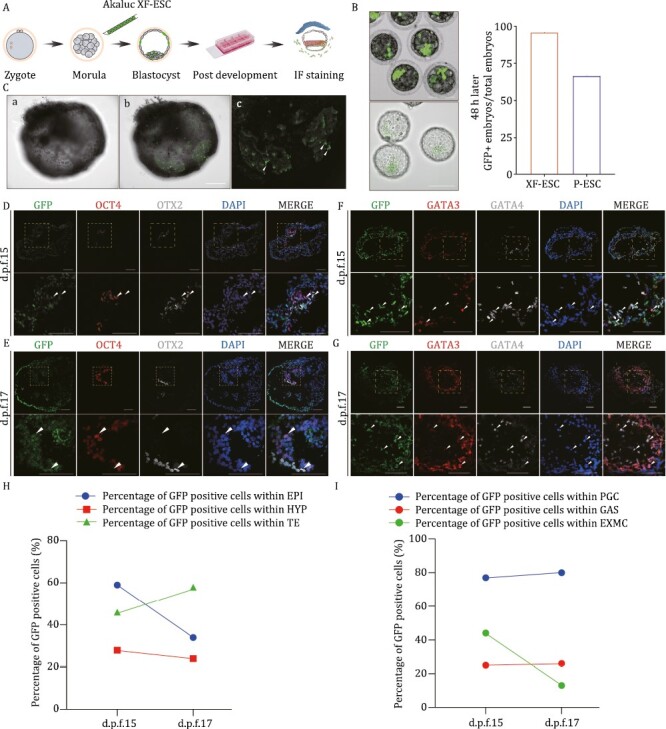
Chimeric contribution of XF-ESCs to peri- and post-implantation monkey embryos. (A) Schematic of the generation and analyses of chimeric embryos derived from blastocyst injection of XF-ESCs. (B) XF-ESCs were injected into embryos (upper); 48 h after XF-ESCs were injected into embryos (lower). Scale bars, 200 μm; The development efficiency of XF-ESC containing chimeric embryos (*n* = 24) and P-ESCs chimeric embryos (*n* = 15) (Kang et al. 2018) was compared 48 h after embryos injection (right). (C) 12 days (d.p.f.18) after XF-ESCs were injected into embryos (*n* = 1) (a, BF image; b, merged image; c, copGFP positive image). Scale bars, 200 μm. The arrow indicates injected cells. (D and E) Representative IF staining images showing the integration of CMV-AkaLuc-EF-1α-copGFP + XF-ESCs into host monkey embryos at d.p.f.15 (*n* = 3) to d.p.f.17 (*n* = 3). The embryos were stained for CMV-AkaLuc-EF-1α-copGFP, OCT4, and OTX2 (scale bar, 100 μm). Higher-magnification images of selected single planes of the boxed areas are shown below (scale bars, 100 μm). Arrows indicate CMV-AkaLuc-EF-1α-copGFP+ XF-ESCs expressing copGFP, OCT4, or OTX2. (F and G) Representative IF staining images showing XF-ESCs differentiated into HYP-like cells and TE-like cells within host monkey embryos at d.p.f.15 (*n* = 3) to d.p.f.17 (*n* = 3). The embryos were stained for GATA4 and GATA3. Scale bar, 100 μm. Higher-magnification images of selected single planes of the boxed areas are shown below (scale bar, 100 μm). Arrow indicates CMV-AkaLuc-EF-1α-copGFP+ XF-ESCs expressing GATA3 or GATA4. (H) Levels of chimerism of XF-ESCs within EPI, HYP, and TE. EPI cells expressed OCT4 or SOX2, and HYP cells expressed GATA6 or GATA4, whereas TE expressed GATA3 (a total of six embryos were analyzed). EPI, epiblast; HYP, hypoblast; TE, trophectoderm. (I) Levels of chimerism of XF-ESCs within PGCLCs, GAS, and EXMC. PGCLCs expressed TFAP2C or SOX17, and GAS cells expressed OTX2, whereas EXMC expressed COL6A1 (a total of six embryos were analyzed). PGCLCs, primordial germ cell-like cells; GAS, gastrulating; EXMC, extra-embryonic mesenchyme cell.

We took advantage of an established prolonged embryo culture system to study the development of homologous chimeric embryos in NHP. The embryo manipulation procedures performed are shown in Fig. 3A. First, we found that chimeric cells could normally grow together with host cells within 48 h after injection (Fig. 3B). Meanwhile, within 48 h of chimera formation, we had a higher rate of GFP+ embryos (Fig. 3B) (Kang et al. 2018). After attachment, the chimeric embryos continued to grow. At d.p.f.18, CMV-AkaLuc-EF-1α-copGFP+ cells were also detected in the monkey embryos (Fig. 3C).

To explore the developmental potential of XF-ESC in monkey embryos, we performed immunofluorescence (IF) for several embryonic and extra-embryonic lineages using specific antibodies to study the developmental potential of XF-ESCs in monkey embryos. At d.p.f.15 to d.p.f.17, these XF-ESCs appeared to undergo differentiation into gastrulating (GAS) cells as evidenced by the increased expression of OTX2 (Vincent et al. 2003; Martyn et al. 2018), while OCT4 or SOX2 expression was maintained (Fig. 3D, 3E and S3D). Meanwhile, CMV-AkaLuc-EF-1α-copGFP+ cells were detected that expressed GATA3, GATA4, and GATA6 from d.p.f.15 to d.p.f.17 (Figs. 3F, 3G, S3H and S3I). Primate PGCLCs are usually formed during the second- or third-week post-fertilization. At d.p.f.15 to d.p.f.17, XF-ESCs were detected that expressed SOX17 and TFAP2C suggesting that they had the ability to differentiate into PGCLCs (Fig. S3D and S3E). FOXA1 and COL6A1 expression were used to delineate the visceral and yolk sac endoderm (VE/YE) (Boroviak and Nichols 2017) and extra-embryonic mesenchyme cells (EXMCs) (Enders et al. 1986). Furthermore, CMV-AkaLuc-EF-1α-copGFP+ cells expressing COL6A1, a marker of EXMCs, was detected, suggesting ongoing differentiation of XF-ESCs toward EXMCs. In addition, CMV-AkaLuc-EF-1α-copGFP+/FOXA1+ cells were detected, suggesting that XF-ESCs can differentiate into VE/YE cells (Fig. S3F and S3G).

Overall, we found that XF-ESCs contributed to the post-implantation EPI (with the highest contribution of 59% observed at d.p.f.15), hypoblast (HYP) (with the highest contribution of 28% observed at d.p.f.15), trophectoderm (TE) (with the highest contribution of 58% observed at d.p.f.17), PGCLC (with the highest contribution of 80% observed at d.p.f.17), GAS (with the highest contribution of 26% observed at d.p.f.17), and EXMC (with the highest contribution of 44% observed at d.p.f.15) (Fig. 3H and 3I). In general, these results suggest that XF-ESCs have the potential to produce chimeric bioluminescent monkeys.

### Generation of a bioluminescent chimeric monkey

**Figure 4.**
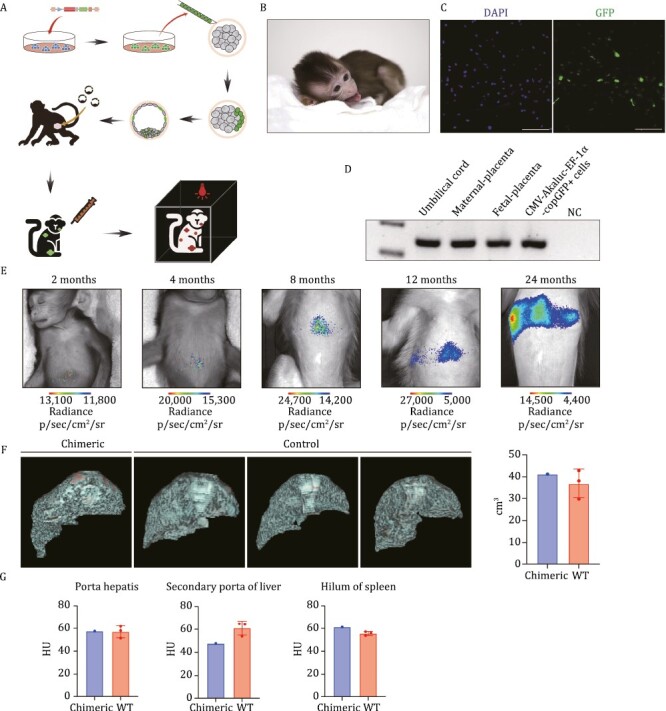
Generation of a bioluminescent chimeric monkey. (A) Schematic representation of the *in vivo* application of bioluminescence and chimeric technology. (B) A representative image of the XF-ESCs-derived chimeric monkey. (C) Representative IF staining images of CMV-AkaLuc-EF-1α-copGFP+ XF-ESCs in the umbilical cord of a chimeric neonatal monkey (Scale bar, 100 μm). (D) Representative gel images of genomic PCR analysis of neonatal monkey using the AkaLuc specific primers. (E) Representative images of the chimeric monkey bearing AkaLuc. (F) Anterior oblique 3D view of the liver. Individual animal liver volume (right), WT, *n* = 3, biological replicates, data are mean with SD, unpaired two-tailed *t*-test, *P* = 0.631. (G) Representative images of Hounsfield units (HU) count liver are shown. The HU scale is a quantitative scale for describing radiodensity in medical CT and provides an accurate density for the type of tissue. WT, *n* = 3, biological replicates, data are mean with SD, unpaired two-tailed *t*-test; Porta hepatis, *P* = 0.941; Secondary porta of liver, *P* = 0.201, Hilum of spleen, *P* = 0.112.

The BLI system composed of AkaLumine and AkaLuc is a bioengineered light source (Iwano et al. 2018) that has high sensitivity, an outstanding signal/noise ratio, and favorable properties for noninvasive *in vivo* imaging (Dothager et al. 2009; Genevois et al. 2016; Mezzanotte et al. 2017). However, we needed to ascertain whether this tool would work in monkeys. To exclude the possibility of background fluorescence from the NaCl solution, we mixed the NaCl solution with AkaLumine and observed no spontaneous fluorescence (Fig. S4A). Additionally, to assess the fluorescence signal detection time of AkaLumine with CMV-AkaLuc-EF-1α-copGFP+ cells *in vitro*, we continuously monitored the mixture of AkaLumine and CMV-AkaLuc-EF-1α-copGFP+ cells for 30 min. We observed that the fluorescence signal was strongest when the cells initially came into contact with AkaLumine (Fig. S4B and S4C). Therefore, we combined purified CMV-AkaLuc-EF-1α-copGFP+ cells with the substrate, then introduced these cells into the monkey using intravenous injection. We found that the cells collected in the chest cavity (Fig. S4D and S4E). We subcutaneously injected 10,000 fluorescent-labeled cells into the abdominal and dorsal regions of wild-type mice. We observed that the fluorescence signal did not penetrate the entire tissue (Fig. S4F). However, when we intraperitoneally injected 1,000,000 cells into mice, we detected fluorescence signals from multiple directions (Fig. S4G). It is important to note that the detection of fluorescence signal decreases as tissue thickness increases (Fig. S4H). Next, in order to validate the luciferase-labeled chimeric monkey, we transplanted monkey chimeric embryos into surrogate monkeys, which were generated and analyzed *in vivo* (Fig. 4A).

To determine long-term integration, we transferred chimeric embryos into surrogate mothers. Seven recipients were confirmed to be pregnant, each carrying one fetus (Fig. S5A). The pregnancy and implantation rates of chimeric blastocysts were 63.6% (7/11) and 44.2% (19/43), respectively. Fortunately, a female monkey baby was born (Fig. 4B), and immunofluorescence staining of the umbilical cord of the baby chimeric monkey showed copGFP+ cells (Fig. 4C). We also detected the presence of the AkaLuc gene by PCR and sequencing (Figs. 4D and S5B). To further validate chimeric identities, we performed bioluminescent imaging of the chimeric monkey. The monkey was first anesthetized, and when we injected luciferin intravenously, the bioluminescence was detected successfully through *in vivo* imaging (Fig. 4E). Meanwhile, the bioluminescence signal in the abdomen could be detected continuously from different directions and we conducted live tracking of chimeric animals for two consecutive years, which is crucial for assessing the viability of cells in primate research (Fig. S6A). Due to previous reports in the literature suggesting spontaneous fluorescence upon injection of AkaLumine into animal models, we aimed to exclude the possibility of spontaneous fluorescence in our chimeric monkey model (Su et al. 2020). To achieve this, we selected wild-type (WT) monkeys of the same age as the chimeric model and administered AkaLumine intravenously based on their body weight. Through continuous observation for 30 min, we did not detect any spontaneous fluorescence (Fig. S6B and S6C). Furthermore, using the same method, we administered AkaLumine intravenously to the chimeric monkey model and obtained consistent results as before (Fig. S6D and S6E).

In order to assess the true background signal in the absence of any reporter enzyme, we conducted direct testing of spontaneous emission from AkaLumine *in vivo*. Specifically, we administered the substrate to WT mice that do not express any reporter, thereby enabling us to accurately evaluate the background signal level (Fig. S7A). By administering the substrate intraperitoneally into mice, we observed spontaneous emission (Fig. S7B), consistent with previous reports. However, when we administered the substrate via subcutaneous injection in the neck region and continuously observed for 8 min, no spontaneous emission was detected (Fig. S7C). Additionally, we observed that the intensity of spontaneous emission increased when AkaLumine was dissolved and kept under light and room temperature conditions for an extended period of time (Fig. S7D). Meanwhile, we found that cells treated with H_2_O_2_, although non-viable, exhibited fluorescence when mixed with AkaLumine (Fig. S7G). We speculate that this may be due to the dependence of AkaLuc luciferase expression on ATP. To further demonstrate that the fluorescence observed in chimeric monkeys is not due to spontaneous emission, we injected the substrate, which had been dissolved and kept under light and room temperature conditions for an extended period of time, into WT monkeys of the same age that do not express any reporter genes. This allowed us to evaluate the true background in the absence of enzyme. In this scenario, we detected results similar to those observed in mice in the chest region of the monkeys (Fig. S7E). By using spontaneous emission as a reference and setting a minimum threshold for signal enhancement, we found that the fluorescence intensity in the chimeric monkey was stronger compared to the spontaneous emission signal in WT monkeys (Fig. S7F). In brief, our results demonstrate that the fluorescence observed in the chimeric monkey model is intentional and genuine and the direct testing of spontaneous emission from AkaLumine *in vivo*, conducted in WT mice and monkeys, confirms the validity of our results. We have successfully tracked the survival of allogeneic cells in a large animal model for over 2 years, providing valuable insights for the field of cell therapy.

The aborted fetuses were examined using the genomic polymerase chain reaction (PCR) assay. We detected AkaLuc sequences in multiple organs (Fig. S8A), and we also detected AkaLuc sequences and copGFP proteins in testis (Fig. S8B). We observed that XF-ESCs contributed to a wide range of tissues and organs in aborted fetuses. Representative copGFP staining showed that CMV-AkaLuc-EF-1α-copGFP+ cells could contribute to multiple organs/tissues, including skin, intestines, heart, and testis in the aborted fetuses (Fig. S8C). Through PCR detection AkaLuc sequences, we found that the chimeric ratio in different tissues ranged (Fig. S8D and S8E).

Next, we investigated the safety of chimeric cells in a monkey model. Computed tomography (CT), a powerful diagnosis and therapy guidance technique, was used to examine organ changes. Evaluation of the data following chimeric monkey and WT monkeys showed no abnormal tissues. The liver volume showed no significant difference compared to that of the WT (Fig. 4F). Meanwhile, the liver density at different layers showed no significant difference between chimeric monkey and WT monkeys (Figs. 4G and S9A). In addition, we selected three WT monkeys of the same age as the chimeric monkey as controls. In addition, we identified a group of 27 WT adult female monkeys, aged between 5 and 7 years, to serve as a broader comparison. Their liver and kidney functions were examined through blood tests at ages one and two. We wondered whether chimeric cells had an effect on monkey liver as a strong signal was observed in the liver through *in vivo* imaging. The results revealed that the various indicators for the chimeric monkey not only resembled those of the same-aged WT monkeys but also fell within the range observed across the 27 WT monkeys (Fig. S10A). Therefore, we conclude that the presence of chimeric cells currently does not impact the health of the monkey. We will continue to monitor health conditions in the future. Therefore, the above analysis found that CT data, liver and kidney function and pathological analysis were all within the normal range. The chimeric cells’ status and the overall health of the chimeric monkey warrant ongoing longitudinal observations and evaluations.

Together, these results demonstrate that we successfully generated a chimeric monkey model with bioluminescence. Additionally, XF-ESC CMV-AkaLuc-EF-1α-copGFP+ cells contributed to all three germ layers and placenta, indicating well-balanced chimerism.

## Discussion

Despite major advances in human and rodent PSC research, establishing monkey ESCs comparable to the mouse and human counterparts is still challenging (Niu et al. 2017; Fu et al. 2020). Organoid derivation and xenotransplantation hold great potential for diverse applications in regenerative medicine as well as for producing human tissues and organs for replacement therapies (Tan et al. 2021). However, noninvasive *in vivo* imaging techniques for studies of large animal organs are limited. Therefore, it is necessary to establish a comprehensive research system to complement human stem cell research and develop therapeutic applications. This system requires efficient PSC culture and maintenance, *in vivo* transplantation, and bioluminescent image tracing. Due to the high similarity between primates and humans, the former has particularly strong potential for supporting clinical research through studying therapeutic issues such as chimeric cell competition (Tan et al., 2021; Zheng et al., 2021; De Los Angeles and Wu, 2022) and blastocyst construction (Rivron et al. 2018; Yu et al. 2021a; Yanagida et al. 2021; Kagawa et al. 2022).

In the course of this study, we have used a dual reporter construct driven by CMV and EF-1α promoters, respectively. While this configuration served our purpose, it is important to recognize potential limitations associated with these choices. Notably, the CMV promoter is known to be susceptible to epigenetic silencing, particularly in the context of cell differentiation. This could potentially contribute to the variability observed in our bioluminescence results, both *in vitro* and *in vivo*. Furthermore, the EF-1α promoter can also undergo epigenetic modifications, which may impact GFP expression levels. We appreciate the significance of these limitations and understand that they may detract from the robustness of our findings. Therefore, in future studies, we plan to employ alternative promoters that are less susceptible to epigenetic silencing.

In this study, the culture system we developed is stable under xeno-free conditions, shows metabolism similar to that of naïve state, and also activates pathways (e.g., MAPK and PI3K-AKT) that may improve chimeric efficiency. Furthermore, the PSCs cultured under this system not only differentiate directly into PGCLCs *in vitro* but also give rise to chimeric cells in different germ layers, including PGCLCs, in post-implantation culture embryos and aborted fetuses. Therefore, we have achieved an NHP model under a new chemically defined and xeno-free culture system, alongside *in vitro* differentiation tests and transcriptome characteristics showing that monkey PSCs can maintain a naïve-like state of pluripotency in this culture system. This result lays the foundation for future research on primate organoids and xenotransplantation. At the same time, the chimerism test will not only verify the pluripotency of stem cells but also help to verify the feasibility of organ compensation in NHPs in the future. Currently, we have examined one chimeric monkey carrying an AkaLuc-reporter gene. While the results from this single subject suggest that *in vivo* tracking at the level of large animals is possible, we recognized the limitations of extrapolating from one case. Therefore, we consider it essential to increase the number of subjects in future studies for more reliable and comprehensive insights. Nevertheless, the ability to monitor bioluminescence in living animals for more than 2 years with our chimeric monkey provides a promising direction for exploring the effectiveness of xenografts. With further validation, real-time *in vivo* imaging could potentially improve the efficiency of transplantation studies.

## Methods

### Cynomolgus monkeys

Healthy male and female cynomolgus monkeys and rhesus monkeys, ranging in age from 5 to 10 years, were selected for use in this study. The cynomolgus monkeys were housed with a 12-h light/dark cycle between 06:00 and 18:00 in a temperature-controlled room (22°C ± 1°C) with free access to water and food.

### Cell cultures

All monkey PSCs were cultured without antibiotics in humidified incubators at 37°C in 5% CO_2_ and maintained in atmospheric oxygen. Cell lines tested negative for mycoplasma by periodic PCR screening.

### Culture of xeno-free monkey PSCs

Xeno-free monkey PSCs were cultured in a chemically defined medium under 20% O_2_ and 5% CO_2_ at 37°C. XF-PSC medium was prepared by including E8 medium supplemented with 1× Chemically Defined Lipid Concentrate (Gibco), 1× Glutamax (Gibco), 1.94 mg/L Glutathione (Sigma), 100 ng/mL of Nodal (MCE), 2 μmol/L IWR-1 (Selleck), and 10 ng/mL of Activin A (Peprotech). Prepared XF-PSC medium could be kept at 4°C for up to 1 week.

XF PSCs were cultured on Vitronectin XF (STEMCELL)—coated plate, which was diluted in Cell adhere dilution buffer (STEMCELL). The final concentration of Vitronectin XF was 50 μg/mL. For culturing XF PSCs in 24-well plates, 350 μL of diluted Vitronectin was added to one well, then the plates were incubated at 37°C for 1.5 h. Do not allow the culture surface to dry as the matrix will become inactivated. For the initial passaging and culturing, Y27632 (STEMCELL) or Clone R (STEMCELL) was needed.

### Culture of primed monkey PSCs

The iPSC, NTESC, and ESC cell lines were established in our laboratory. Conventional primed monkey PSCs were cultured in DMEM/F12 (Thermo Fisher Scientific) with 15% KSR (Gibco) containing 10 ng/mL bFGF (Peprotech), 0.1 mmol/L β-Me (STEMCELL), NEAA (Thermo Fisher Scientific), and 20% PSGro® Human iPSC/ESC Growth Medium (StemRD). All monkey-primed PSC lines were cultured on mitomycin-inactivated CF-1 mouse embryonic fibroblasts in PSC growth media.

### Conversion of conventional PSCs into XF PSCs

To digest primed monkey PSCs for conversion, the conventional monkey PSC medium was removed from the wells, and XF-PSC medium was used to wash to ensure that no dead cells or cell debris remained in the culture. Then, primed monkey PSCs were dissociated with Accutase (STEMCELL) and seeded to Vitronectin XF-coated plate in appropriate volume (according to the cell lines and growth ratio). To increase viability and proliferation rate, passaging at a high density (1:1–2 ratio) was preferred for the first three passages. In our hand, the conversion was approximately taken about 5–10 passages, then XF-PSCs could be propagated well in XF PSC medium.

### Reprogramming fibroblasts into iPSCs

Monkey fibroblast cells were isolated from skin and further cultured in fibroblast culture medium, which contained DMEM (Thermo Fisher Scientific) plus 15% fetal bovine serum (FBS, Thermo Fisher Scientific) and 1% minimum essential medium (MEM) non-essential amino acids solution (Thermo Fisher Scientific).

To establish XF-iPSC lines, monkey fibroblast cells were seeded at 1 × 10^5^ cells in 6-well plates and further cultured for 1–2 days in a fibroblast culture medium. The Sendai virus (Thermo Fisher Scientific) was applied to reprogram monkey fibroblast cells. Sendai virus transduction was performed according to the user’s manual. 7 days post transduction, infected monkey fibroblast cells were replated onto Vitronectin XF-coated 6-well plates at 1 × 10^5^ cells/cm^2^. The cultured medium was refreshed to XF-PSC medium. From 7 to 14 days post transduction onwards, XF-iPSC colonies emerged and could be picked up. Colonies were enzymatically dissociated into single cells using Accutase.

### Karyotype analysis

Cells were collected at a density of 60%–80% of confluence on the day of sampling. After 2 h of incubation with fresh medium, colcemid solution was added to the culture at a final concentration of 0.002 mg/mL. Then the cells were incubated for 1 h. After incubation, cells were washed, digested, and centrifuged. To obtain a single-cell suspension, the pellet was resuspended in hypotonic solution (0.56% KCl), and incubated at room temperature for 6 min. After centrifuging and removing the hypotonic solution, 5 mL of ice-cold fixative (3:1 methanol:acetic acid) was added to the suspension in a dropwise manner. Then the cells were incubated at room temperature for 5 min before spinning down. The fixing procedure was repeated for additional three times. Afterwards, the pellet was resuspended in a final volume of 1 mL fixative. Then, the cells were dropped onto 5% acetic acid ± ethanol (ice-cold) washed slides and stained with Giemsa. For each experiment, 30–40 metaphases were analyzed. The number of chromosomes and the presence of structural chromosomal abnormalities were examined.

### PGCLC differentiation

Three thousand cells were plated in low-binding 96-well plates in GK15 medium (GMEM and 15% Knockout Serum Replacement (Gibco), 0.1 mmol/L NEAA (Gibco), 1 mmol/L Sodium Pyruvate (Gibco), 2 mmol/L L-Glutamine (Gibco), 0.1 mmol/L 2-mercaptoethanol (Gibco) supplemented with 500 ng/mL BMP4 (R&D), 100 ng/mL hSCF (R&D), 0.1 mg/mL hLIF (Peprotech), and 50 ng/mL EGF (Peprotech) in the presence of 10 mmol/L Rho-associated kinase inhibitor Y27632.

### EB formation assay

Cells were dissociated into single cells and cultured for 7 days on ultra-low attachment 6-well plates in IMDM (Gibco) supplemented with 15% FBS at a density of 5 × 10^5^ per well. To increase cell viability, 5 μmol/L of Y27632 was added to the medium for the first 24 h. Medium change was performed every 2 days. Then, EBs were collected and plated on the Matrigel-coated 24-well plates for another 7 days in the same medium. Then cells were fixed for analysis.

### Teratoma assay

Approximately 5 × 10^6^ xeno-free PSCs were suspended in 50 μL XF-PSC medium, and mixed with the same volume of Matrigel (thawed before the experiment on ice). The cell mixture was subcutaneously injected into immunodeficient NOD/SCID mice. Teratomas developed within 4–8 weeks. The teratomas were isolated and embedded in paraffin, which were processed for hematoxylin and eosin staining.

### Genomic PCR

Genomic PCR was used to detect the chimerism of various tissues in the monkey. Genomic DNA was extracted using the high-intensity salt precipitation method. 50 ng of genomic DNA for each sample was used for PCR. Genomic PCRs were performed with Premix TaqTM (TaKaRa TaqTM Version 2.0 plus Dye) polymerase. AkaLuc-1-OF forward primer (5ʹ-AGGACGCCAAGAACATCAAG-3ʹ), AkaLuc-1-OR reverse primer (5ʹ-CTTCTTGCTCACGAACACCA-3ʹ). Nested PCR was used for target band amplification. In the first round, 20 cycles of PCR were performed at Tm 59°C. The second round used 1 μL of first-round PCR products as templates, and the PCR was run at Tm 59°C for 30 cycles. AkaLuc-1-IF forward primer (5ʹ-ACGCCGAGTACTTCGAGATG-3ʹ), AkaLuc-1-IR reverse primer (5ʹ-CTTCTTGCTCACGAACACCA-3ʹ).

### Statistical analyses

When two groups were compared, a two-tailed Student’s *t*-test was used to assess statistical significance. Data are presented as mean with SD and mean with SEM. All data were analyzed using GraphPad 9.0 software. Quantification was performed by Image J, and a *P* ≤ 0.05 was considered statistically significant.

### Alkaline phosphatase staining

Cells were fixed in 4% (*w*/*v*) paraformaldehyde (PFA) for 15 min at room temperature. Alkaline phosphatase (AP) substrate solution (Abcam) was prepared per the manufacturer’s instructions. The cells were incubated with AP substrate at room temperature for 15–20 min in the dark.

### Cell population doubling time

The cell population doubling time was calculated using the doubling time online calculator.

### Imaging and quantification of XF-ESCs in monkey using the AkaLuc bioluminescence

Tokeoni (a luciferin analog) was dissolved in saline solution (60 mmol/L) and injected intravenously into the monkeys (75 nmoL/g, Tokeoni). Immediately after the addition of Tokeoni, the monkeys were imaged using an IVIS Spectrum (PerkinElmer) continuously. Bioluminescence was quantified using region of interest (ROI) analysis of the individual wells. The average signal, expressed as the total number of photons emitted per second (photons/s), from each of the wells was calculated using the Living Image 3.2 software (PerkinElmer).

All imaging experiments required anesthesia in monkeys. Prior to anesthesia, a 12-h fasting period was implemented, and intramuscular injections of atropine sulfate solution (0.05 mg/kg) and ZoletilTM (0.05 mg/kg) were administered. The experiments were initiated once the monkeys were observed to be in a normal physical condition after the injections. Following the completion of the experiments, intramuscular injections of xylazine hydrochloride solution (0.025 mL/kg) were administered. This approach typically maintained the anesthesia state for approximately one hour. Imager settings (Table S2).

### CT scan

CT scanners were used as Optima CT680 (GE Healthcare). The characteristics of CT scanning: tube voltage of 120 kV, rotation (0.6 s), matrix (512), pitch (0.985꞉1), slice thickness (2.5 mm), and reconstruction slice thickness (0.625 mm). Liver volume was analyzed using the Hepatic VCAR 15.0 ext8 software. Skeletal reconstruction was performed using Volume Rendering software, and all reconstructions were automatically analyzed by the software.

### Quantitative image analysis of CT scan

Hounsfield unit (HU) values of the porta hepatis, secondary porta of the liver and hilum of spleen on the nonenhanced phase images were collected. The HU value was identified by manually drawing a circle ROI appropriately within the parenchyma on each phase CT image. The ROI was placed carefully to artifact areas and then copied and pasted onto the other phase of the same area, with suitable placement modification performed as necessary. All measurements were repeated three times, and average values were calculated and used for further analysis.

### Oocyte collection and *in vitro* fertilization

Ovarian stimulation, oocyte recovery and *in vitro* fertilization were performed as previously described (Tan et al. 2021). In brief, healthy female cynomolgus monkeys were subjected to follicular stimulation by intramuscular injection of 20 IU of recombinant human follitropin alpha (rhFSH, Gonal F, Merck Serono) for 8 days, then 1,000 IU recombinant human chorionic gonadotropin alpha (rhCG, OVIDREL, Merck Serono) was injected on day 9. Cumulus-oocyte complexes were collected by laparoscopic follicular aspiration 32–35 h following rhCG administration. Follicular contents were placed in HEPES-buffered Tyrode’s albumin lactate pyruvate (TALP) medium containing 0.3% bovine serum albumin (BSA) at 37°C. Oocytes were stripped of cumulus cells by pipetting after a brief exposure (<1 min) to hyaluronidase (0.5 mg/mL) in TALP-HEPES to allow visual selection of nuclear maturity meta-phase II (MII; first polar body present) oocytes. The mature oocytes were subjected to intracytoplasmic sperm injection (ICSI) immediately and then cultured in CMRL-1066 medium (Gibco) containing 10% FBS, at 37°C in 5% CO_2_. Fertilization was confirmed by the presence of the second polar body and two pronuclei. Zygotes were then cultured in the chemically defined hamster embryo culture medium-9 (HECM-9) containing 10% FBS at 37°C in 5% CO_2_ to allow embryo development. All chemicals were from Sigma Chemicals unless otherwise stated.

### Microinjection of XF-ESCs into monkey blastocysts

Normal developed morulae (early stage blastocyst) were transferred into a 50 mL manipulation droplet of TH3 in the center of a Petri dish covered with 3 mL of mineral oil. Single-cell suspensions of AkaLuc labeled ESCs were placed into a separate 10 μL droplet of culture medium next to the manipulation drop. Tensingle copGFP positive XF-ES cells were aspirated into a 15 mm inside diameter injection pipette with 30°C oblique mouth. The blastocyst was held with a holding pipette and the injection pipette was moved to the manipulation droplet. Meanwhile, the zona pellucida was ablated using a single laser pulse, and the injection pipette containing XF-ES cells was immediately inserted into the hole in the blastocyst, close to the ICM. Injected blastocysts were quickly transferred into the mixed media of HECM-9 and XF-PSC culture media (1:1).

### *In vitro* embryo culture

Chimeric blastocysts were treated with acidic Tyrode’s solution to remove the zona pellucida and transferred to an ibiTreat 8-well μ-plate (Ibidi) containing 300 μL of pre-equilibrated *in vitro* culture medium 1 (IVC1). On the second day, 150 μL of IVC1 was carefully removed and 200 μL pre-equilibrated *in vitro* culture medium 2 was added. Blastocyst growth was monitored and the medium was changed every 2 days until the termination of experiments.

### Embryo immunofluorescence analysis

Monkey post-implantation embryos cultured *in vitro* were harvested at different stages from day 15 to day 19, and whole embryos were fixed in 4% paraformaldehyde in PBS for 30 min at 25°C. Then embryos were washed 3–5 times with PBS. Embryos were dehydrated in sucrose (Meilunbio) solutions each for 6 h with increasing concentration from 15% to 30% [15%, 20%, 30%; (*w*/*v*)]. Next, embryos were embedded in OCT, frozen and stored at −20°C. Then the samples were prepared as cryosections with 10 μm thickness on pretreated glass slides (CITOTEST), and air-dried for 1 h. After permeabilized in PBST (PBS with 0.3% Triton X-100) (Sigma) for 30 min at 25°C, samples were blocked with 3% (*w*/*v*) BSA in PBS overnight at 4°C. Slides were then incubated with primary antibodies overnight at 4°C. After washing, fluorescence-conjugated secondary antibodies and 4', 6-diamidino-2-phenylindole (DAPI) were incubated with the slides in the dark at 25°C for 2 h. Images were taken using Leica TCS SP8 confocal microscope (Leica) and AX (Nikon).

### Cell immunofluorescence analysis

Cells were fixed on plates in 4% PFA for 15 min at RT. Cells were permeabilized and blocked with 3% BSA/PBS 0.1% Triton X-100. Primary and secondary antibodies were incubated for 2 h at RT or overnight at 4°C.

Embryo transfer and pregnancy diagnosis. Female monkey recipients with proper hormone levels of β-estradiol and progesterone were used as surrogate recipients. Each recipient received two to four blastocysts. The pregnancy was primarily diagnosed by ultrasonography at 2–3 weeks after embryo transfer. Clinical pregnancy and the number of fetuses were confirmed by fetal cardiac activity and the presence of gestation sacs.

### Transcriptome and methylome analysis

For transcriptomics data, raw reads from RNA sequencing data in this study and previous studies (Chen et al. 2015) were subjected to adaptor trimming and filtering of low-quality reads by fastp (v0.21.0) (Chen et al. 2018). Qualified reads were mapped to the cynomolgus monkey reference genome (5.0.91) using Hisat2 (v2.1.0) (Kim et al. 2019). Gene expression was inferred from BAM files using stringtie (v2.0.4) (Pertea et al. 2015) and reported as transcripts per million. Principal component analysis was performed using prcomp function in R language with parameters “center = TRUE” and “scale = TRUE” setting. Differentially expressed genes were analyzed using the R package “limma” (v 3.44.3) (Ritchie et al. 2015). Functional enrichment analysis was performed using the function “enrichr” in the R package clusterProfiler (Yu et al. 2012). The heatmap showing the gene expression was drawn using R package pheatmap (v1.0.12). Calculate the correlation of our data separately with GSE74767 and GSE61420. The batch effect between data was removed using the ComBat function in package sva (v3.40.0) with the parameters “mean.only = T” setting. The correlation between the data was calculated using the cor function with parameters “method = ‘pearson’”. The heatmap showing correlation was drawn using R package pheatmap (v1.0.12). The other graphs were created and visualized using R package ggplot2 (v3.3.2).

For whole genome bisulfite sequencing (WGBS) data, raw reads of WGBS data were trimmed to remove reads containing adapters, low-quality and poly-N reads by fastp (v0.21.0) (Chen et al. 2018). Then, the clean paired-end WGBS reads for each sample were aligned to the cynomolgus monkey reference genome (5.0.91) using BS-Seeker2 (v2.1.7) (Guo et al. 2013) with default settings. Duplicate reads produced during PCR amplification were discarded using sambamba (Tarasov et al. 2015). DNA methylation levels for each cytosine were then calculated by using bs_seeker2-call_methylation.py with default parameters. The global DNA methylation distribution of mC in different target sequences (mC, mCG, mCHG, and mCHH) and the average methylation levels of the target region (chromosome, gene body, and exon) were calculated by CGmaptools (Guo et al. 2018) and visualized using R package ggplot2 (v3.3.2).

## Supplementary Material

pwad049_suppl_Supplementary_Figures_S1-S10_Tables_S1-S2

## Data Availability

The main data supporting the results in this study are available within the paper and its Supplementary Information. The sequence data reported in this paper have been deposited in the Genome Sequence Archive in the National Genomics Data Center, Chinese Academy of Sciences, under accession number CRA009731 that are publicly accessible at the website of China National Center for Bioinformation. All the experimental materials generated in this study are available from the corresponding authors upon reasonable request.

## Code availability

Codes are available from the corresponding authors upon reasonable request.
