# Supplementary material for: Long-term in vivo chimeric cells tracking in non-human primate
Source: Protein Cell. 2023 Sep 27;15(3):207–22. doi: 10.1093/procel/pwad049 (PMC10903985; doi:10.1093/procel/pwad049)
Supplement: pwad049_suppl_Supplementary_Figures_S1-S10_Tables_S1-S2 [file pwad049_suppl_supplementary_figures_s1-s10_tables_s1-s2.pdf]

Figure S1. Establishment of xeno-free culturing conditions for XF-ESC lines and XF-iPSC lines from monkey preimplantation embryos and fibroblasts. (A) Scheme depicting strategy for conducting a screen to maintaining pluripotency in xeno-free culturing conditions. (B) The effect of different combinations of Activin-A and IWR-1-endo at varying concentrations on the expression of the ESRRB gene. n = 3 biologically independent samples. Error bars, mean ± S.E.M. Statistical significance was analysed using one-way ANOVA. (C) The effect of different concentrations of Vitronectin on the expression of the POU5F1, NANOG and ESRRB gene. n = 3 biologically independent samples. Error bars, mean ± S.E.M. Statistical significance was analyzed using one-way ANOVA. (D) Morphology of monkey XF-NTESC and XF-iPSC colonies before (a) and after (b) conversion using the XF-PSC medium. Scale bars, 100 μm. (E) Immunostaining of pluripotency marker gene expression in monkey XF-NTESC, XF-iPSC and XF-ESC after conversion using the XF-PSC medium. For each cell line, similar results were obtained in three independent experiments. Scale bar, 100 μm. (F) Representative H&E staining images showing that teratomas formed by xeno-free culture PSCs contained tissues from all three embryonic germ layers. For each cell line, similar results were obtained in two independent experiments. Scale bars, 100 μm. (G) Representative IF images showing differentiation of xeno-free culture PSCs into mesoderm (SMA), and endoderm (AFP, FOXA2), ectoderm (Tubulin-3) lineages. For each cell line, similar results were obtained in two independent experiments. Scale bars, 100 μm. (H) Karyotype analysis of XF-ESCs and XF-iPSCs.

Figure S2. Expansion of pluripotency features from XF-ESCs. (A) Immunostaining of KLF17, TBX3, OCT4, SOX2 and NANOG marker gene expression in monkey ESCs after conversion using the primed PSC condition. For each cell line, similar results were obtained in two independent experiments. Scale bar, 100 µm. (B) Immunostaining of KLF4 and DPPA3 marker gene expression in monkey ESCs after conversion using the primed PSC condition. Similar results were obtained in two cell lines independent experiments. Scale bar, 100 µm. (C) RT-qPCR analysis of XIST and pluripotency markers TFCP2L1, KLF17 and DPPA3. n = 3 biologically independent samples. Error bars, mean ± S.E.M., unpaired two-tailed t-test. (D) Expression level (RNA-seq) of KLF4, DNMT3A and DNMT3B; mean ± S.E.M., unpaired two-tailed t-test. (E) Principal component analysis (PCA) of global gene expression (RNA-seq) of XF-ESCs, P-ESCs. (F) Heat map showing the differentially expressed genes (DEGs) catalogued in XF-ESCs and P-ESCs. (G) KEGG enrichment analyses of the DEGs. Red and blue represent enrichment p values for up- and downregulated DEGs, respectively. (H) Heat map of the correlation coefficients among XF-ESCs, P-ESCs, and reported by GSE61420.

Figure S3. Chimeric contribution of XF-ESCs to peri- and post- implantation monkey embryos. (A) Schematic of the CMV-AkaLuc-EF-1α-copGFP lentivirus vector. (B) Morphology of different passages XF-ESCs. Scale bar, 100 μm. (C) Immunostaining of copGFP, OCT4 and NANOG marker gene expression in monkey XF-ESCs. Passage > 60, Scale bar, 100 μm. (D and E) Representative IF images showing the embryos were stained for SOX17 (gray) and TFAP2C (red) at d.p.f.15 (n=3)-d.p.f.17 (n=3). Scale bar, 100 mm. Higher-magnification images of selected single planes of the boxed areas are shown below. Scale bar, 100 μm. Arrow indicates copGFP-positive XF-ESCs expressing SOX17 and TFAP2C. (F and G) Representative IF images showing the embryos were stained for COL6A1 (gray) and FOXA1 (red) at d.p.f.15 (n=3)-d.p.f.17 (n=3). Scale bar, 100 mm. Higher-magnification images of selected single planes of the boxed areas are shown below. Scale bar, 100 μm. Arrow indicates copGFP-positive XF-ESCs expressing COL6A1 or FOXA1. (H and I) Representative IF images GATA6 (gray) and GATA3 (red) in d.p.f.15 (n = 3) to d.p.f.17 (n = 3) monkey embryos. Scale bar, 100 μm.

Figure S4. Analysis of the relationship between cell number and fluorescence intensity at different tissue thicknesses. (A) NaCl solution and the mixture of NaCl and AkaLumine exhibit no spontaneous fluorescence. The experiment was repeated independently two times with similar results. (B) The decay of bioluminescence signals AkaLumine/AkaLuc of CMV-AkaLuc-EF-1α-copGFP+ cells after the addition of AkaLumine. Measurements were carried out in quintuplicate; data are presented as the mean ± S.E.M (n = 3). (C) Decay of signal over time of AkaLuc signal with AkaLumine substrates; data are presented as the mean ± S.E.M (n = 3). (D) Representative images of cells bearing AkaLuc are shown. (E) Representative images of bearing CMV-AkaLuc-EF-1α-copGFP XF-ES cells (1,000,000 cells) injected monkey are shown. (F) Mouse before injection with CMV-AkaLuc-EF-1α-copGFP-carrying cell line (left); Subcutaneous injection of CMV-AkaLuc-EF-1α-copGFP-carrying cells in the dorsal region (middle); Subcutaneous injection of CMV-AkaLuc-EF-1α-copGFP-carrying cells in the abdominal region (right), n = 3. (G) 1,000,000 cells were intraperitoneally injected into mice, we were able to detect fluorescence signals both facing the abdominal region (left) and facing the dorsal region (right), n = 3. (H) Tissue thickness significantly affects the detection of fluorescence signals. n = 3, unpaired two-tailed t-test.

Figure S5. XF-ESCs integrate into tissues. (A) Developmental and positive efficiencies of XF-ESCs after chimeric operation with IVF embryos *in vivo*. (B) Representative AkaLuc sequencing data. Representative PCR products from the chimeric monkey were randomly chosen to sequence. The sequencing analysis shows that the AkaLuc-PCR products from chimeric monkey have the same sequence as the AkaLuc sequence in the CMV-AkaLuc-EF-1α-copGFP lentivirus vector.

Figure S6. Generation of a bioluminescent chimeric monkey. (A) Representative images of chimeric monkey bearing AkaLuc are shown. (B) No significant luminescence was observed for the wild-type monkey injected with AkaLumine. (C) No significant luminescence was continuous observation for 30 min for the wild-type monkey injected with AkaLumine. (D) No significant luminescence was observed for the chimeric monkey without AkaLumine. (E) Significant luminescence was continuous observation for 30 min for the chimeric monkey injected with AkaLumine.

Figure S7. Details of background and signal comparisons. (A) No significant luminescence was observed for the wild-type without AkaLumine, n=3. (B) Signal were observed with AkaLumine injected. The experiment was repeated independently two times with similar results. (C) No spontaneous fluorescence signal was detected upon continuous imaging of wild-type mice following subcutaneous injection of AkaLumine, n=3. (D) An equal volume of AkaLumine solution was injected into the abdominal region of mouse, only one mouse (middle) exhibited spontaneous fluorescence. The same result was obtained in consecutive imaging sessions, demonstrating consistent findings. (E) Upon injection of light-exposed and room temperature-stored Akalumine into wild-type monkeys, a minimal signal was detected (left). In contrast, when AkaLumine, which was protected from light, stored at -20 degrees Celsius, and rapidly injected after dissolution, was administered to chimeric monkeys, signals were detected in different locations (right). (F) Adjusting the minimum signal threshold until the spontaneous fluorescence in wild-type diminishes, this parameter can still detect bioluminescent signals in the chimeric model. (G) Cells without the bioluminescent reporter gene did not show any detectable signal (left), while the addition of an equal volume of H2O2 resulted in a detectable signal (right).

Figure S8. PCR analysis indicates the presence of chimeric cells in tissues. (A) Representative gel images of genomic PCR analyses of aborted fetuses tissue using the AkaLuc specific primers. (B) Representative immunofluorescence images showing integrated CMV-AkaLuc-EF-1α-copGFP+ cynomolgus monkey cells and co-expressed testis marker (SOX9). Scale bar, 100 μm. (C) Representative immunofluorescence images showing integrated CMV-AkaLuc-EF-1α-copGFP+ cynomolgus monkey cells. Scale bar, 100 μm. (D) Representative quantitative genomic PCR analysis of cynomolgus monkey DNA in the tissues of chimeric monkey derived from blastocyst injection with XF-ESCs. A series of chimeric monkey cell dilutions (1:1000–1:100,000) were run in parallel to estimate the degree of monkey cell integration. (E) Representative quantitative genomic PCR analysis of fetus DNA in the tissues of fetus derived from blastocyst injection with XF-ESCs. A series of chimeric monkey cell dilutions (1:1000–1:100,000) were run in parallel to estimate the degree of monkey cell integration.

Figure S9. Quantitative liver CT analysis of wild- type monkey and chimeric monkey. (A) CT image shows the CT Hounsfield unit (HU) value of the liver. The HU value was identified by manually drawing a circle (yellow) or ovoid ROI on each phase CT image. The ROI was placed carefully and then copied and pasted onto the other phase of the same area, with suitable placement modification performed as necessary.

Figure S10. Blood biomarkers to check the effects of chimeric cells on animal health. (A) Blood biochemical assessment of the effects of chimeric cells on animal health. WT, n = 3 animals per group, repeat 3 times and averaged; Chimeric, n = 1 animals per group, repeat 3 times and averaged; data are mean with SD, unpaired two-tailed t-test. ALP, alkaline phosphatase; TP, total protein; GGT, gamma-glutamyl transferase; AST, aspartate aminotransferase; GLOB, globulin; ALB, albumin; LDH, lactate dehydrogenase; ALT, alanine aminotransferase; GLU, glucose; Cr, creatinine; UA, Uric acid; APOA1, Apolipoprotein AI; TG, triglycerides; HDL-CH, high-density lipoprotein cholesterol; CHOL, total cholesterol; LDL-CH, low density lipoprotein cholesterol; APOB, Apolipoprotein B; TBIL, total bilirubin; DBIL, direct bilirubin; IBIL, indirect bilirubin.

Figure S1

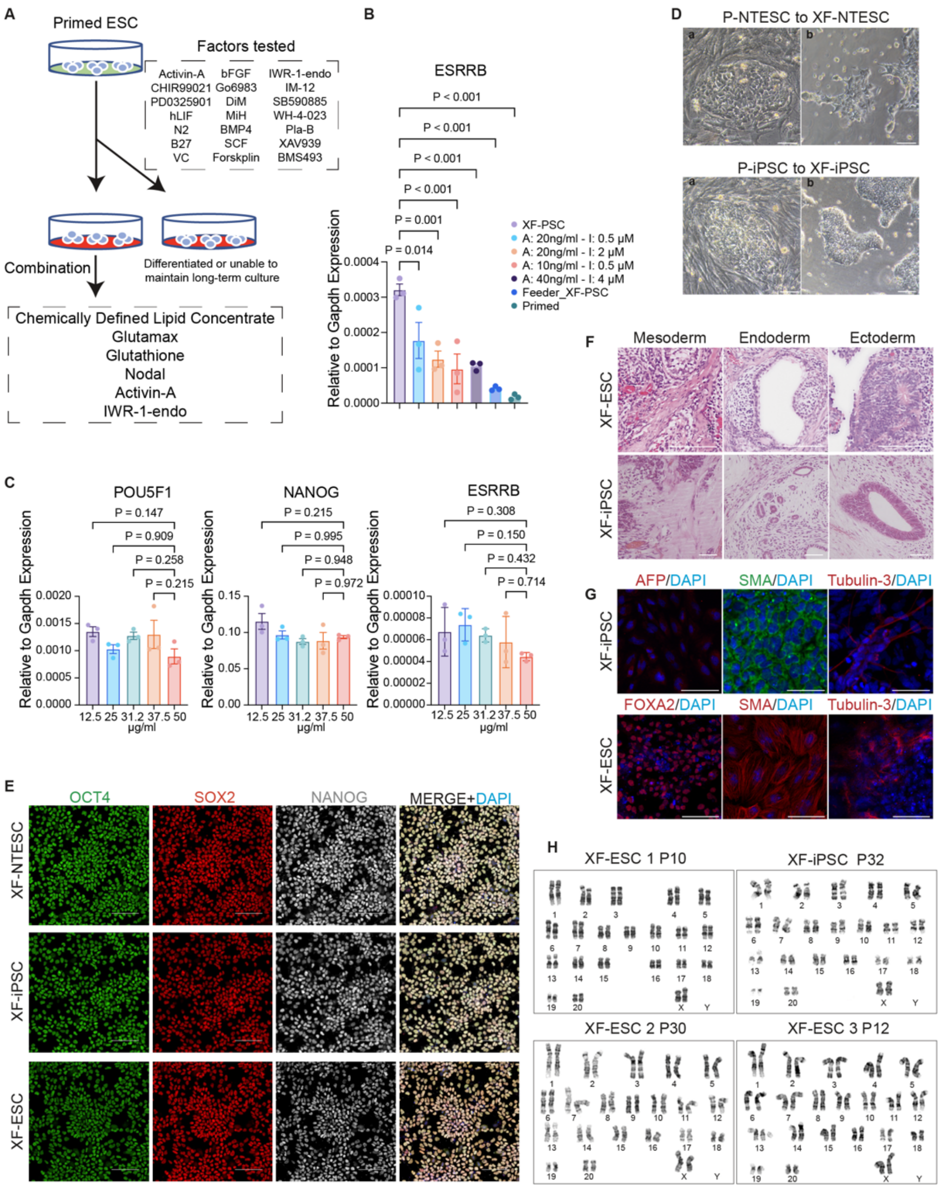

A
Primed ESC
Factors tested
Activin-A bFGF IWR-1-endo
CHIR99021 Go6983 IM-12
PD0325901 DiM SB590885
hLIF MiH WH-4-023
N2 BMP4 Pla-B
B27 SCF XAV939
VC Forskplin BMS493
Combination
Differentiated or unable to maintain long-term culture
Chemically Defined Lipid Concentrate
Glutamax
Glutathione
Nodal
Activin-A
IWR-1-endo
B
ESRRB
P < 0.001
P < 0.001
P < 0.001
P < 0.001
P = 0.001
P = 0.014
Relative to Gapdh Expression
XF-PSC
A: 20ng/ml - I: 0.5 μM
A: 20ng/ml - I: 2 μM
A: 10ng/ml - I: 0.5 μM
A: 40ng/ml - I: 4 μM
Feeder_XF-PSC
Primed
C
POU5F1
P = 0.147
P = 0.909
P = 0.258
P = 0.215
NANOG
P = 0.215
P = 0.995
P = 0.948
P = 0.972
ESRRB
P = 0.308
P = 0.150
P = 0.432
P = 0.714
12.5 25 31.2 37.5 50
μg/ml
D
P-NTESC to XF-NTESC
P-iPSC to XF-iPSC
E
OCT4
SOX2
NANOG
MERGE+DAPI
XF-NTESC
XF-iPSC
XF-ESC
F
Mesoderm
Endoderm
Ectoderm
XF-ESC
XF-iPSC
G
AFP/DAPI
SMA/DAPI
Tubulin-3/DAPI
FOXA2/DAPI
H
XF-ESC 1 P10
XF-iPSC P32
XF-ESC 2 P30
XF-ESC 3 P12

Figure S2

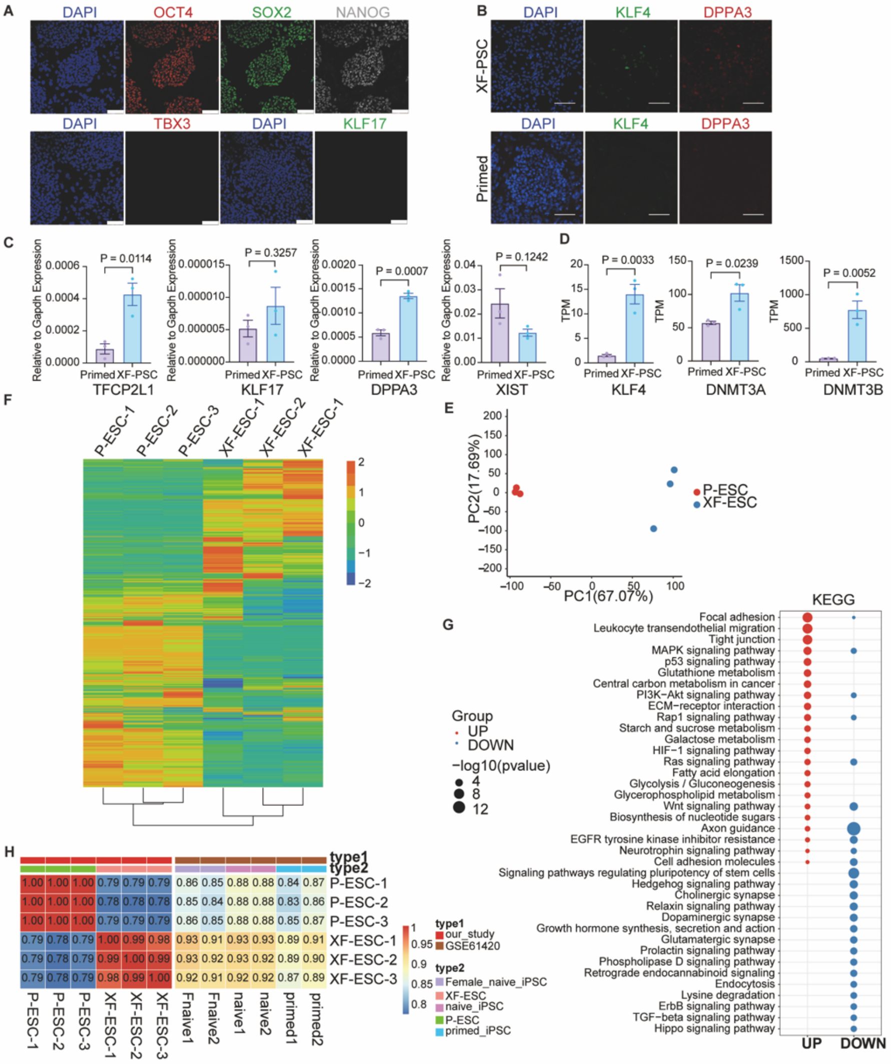
A
DAPI
OCT4
SOX2
NANOG
TBX3
KLF17
B
XF-PSC
Primed
KLF4
DPPA3
C
Relative to Gapdh Expression
P = 0.0114
P = 0.3257
P = 0.0007
P = 0.1242
Primed XF-PSC
TFCP2L1
KLF17
DPPA3
XIST
D
TPM
P = 0.0033
P = 0.0239
P = 0.0052
KLF4
DNMT3A
DNMT3B
F
P-ESC-1
P-ESC-2
P-ESC-3
XF-ESC-1
XF-ESC-2
XF-ESC-1
E
PC2(17.69%)
PC1(67.07%)
P-ESC
XF-ESC
G
KEGG
Group
UP
DOWN
−log10(pvalue)
Focal adhesion
Leukocyte transendothelial migration
Tight junction
MAPK signaling pathway
p53 signaling pathway
Glutathione metabolism
Central carbon metabolism in cancer
PI3K−Akt signaling pathway
ECM−receptor interaction
Rap1 signaling pathway
Starch and sucrose metabolism
Galactose metabolism
HIF−1 signaling pathway
Ras signaling pathway
Fatty acid elongation
Glycolysis / Gluconeogenesis
Glycerophospholipid metabolism
Wnt signaling pathway
Biosynthesis of nucleotide sugars
Axon guidance
EGFR tyrosine kinase inhibitor resistance
Neurotrophin signaling pathway
Cell adhesion molecules
Signaling pathways regulating pluripotency of stem cells
Hedgehog signaling pathway
Cholinergic synapse
Relaxin signaling pathway
Dopaminergic synapse
Growth hormone synthesis, secretion and action
Glutamatergic synapse
Prolactin signaling pathway
Phospholipase D signaling pathway
Retrograde endocannabinoid signaling
Endocytosis
Lysine degradation
ErbB signaling pathway
TGF−beta signaling pathway
Hippo signaling pathway
H
type1
type2
our_study
GSE61420
Female_naive_iPSC
XF-ESC
naive_iPSC
P-ESC
primed_iPSC
Fnaive1
Fnaive2
naive1
naive2
primed1
primed2

Figure S3

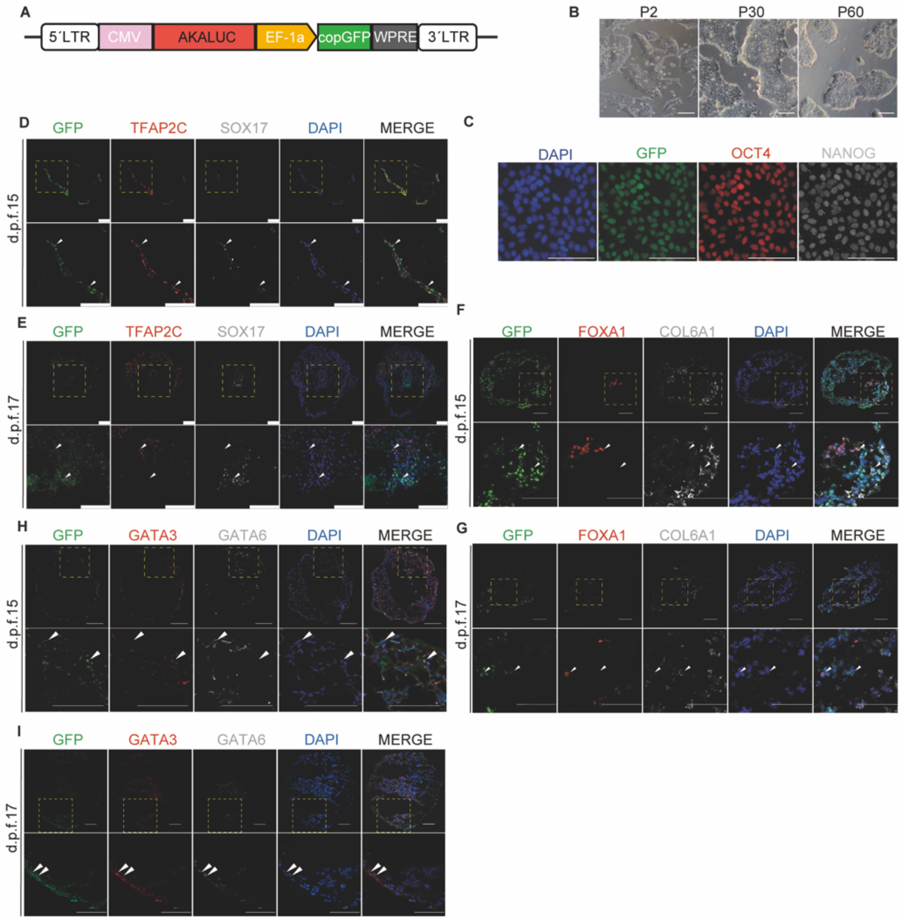

144
145

Figure S4

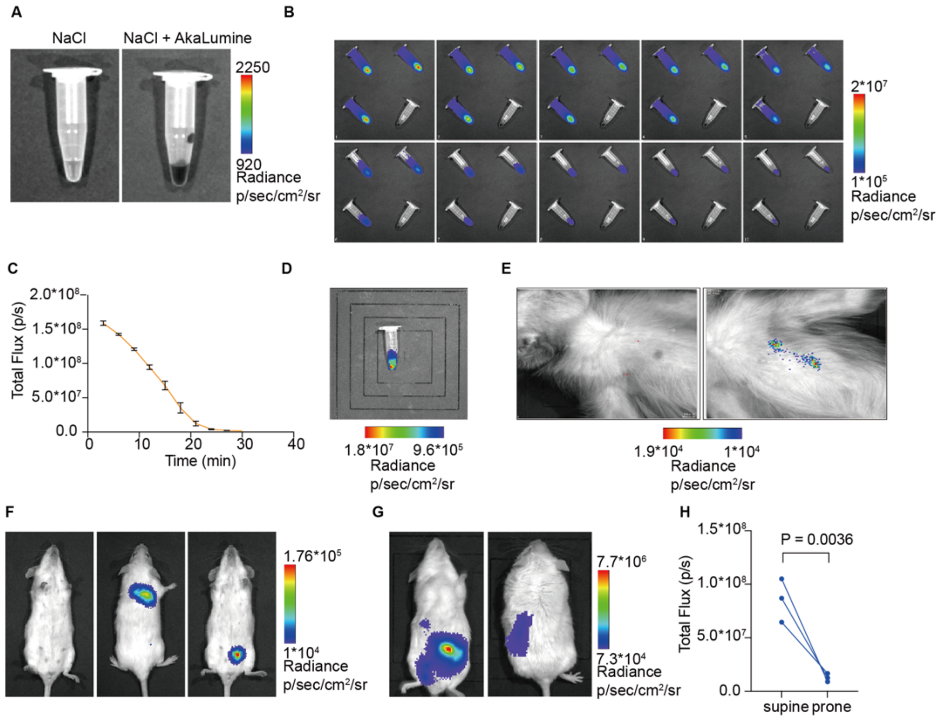

146
147

Figure S5

A

Developmental and positive efficiencies of XF-ESC cells after chimeric operation with IVF embryos in vivo

Donor cell type	Sex	Injected ES cell number/embryo	NO. Embryos transferred	NO. Surrogate mother	NO. Pregnancy	Fetus	Live pups
XF-ESC	Male	10	43	11	7	19	1

B

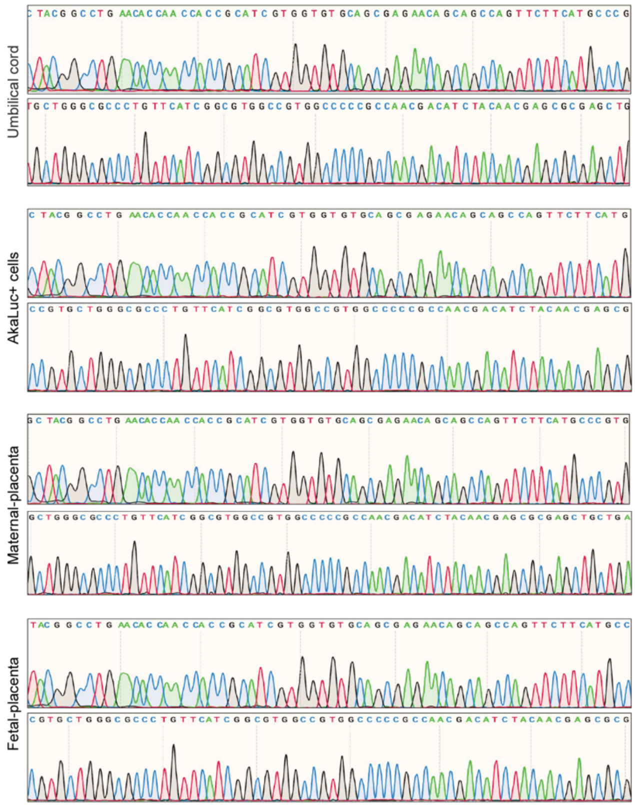

Figure S6

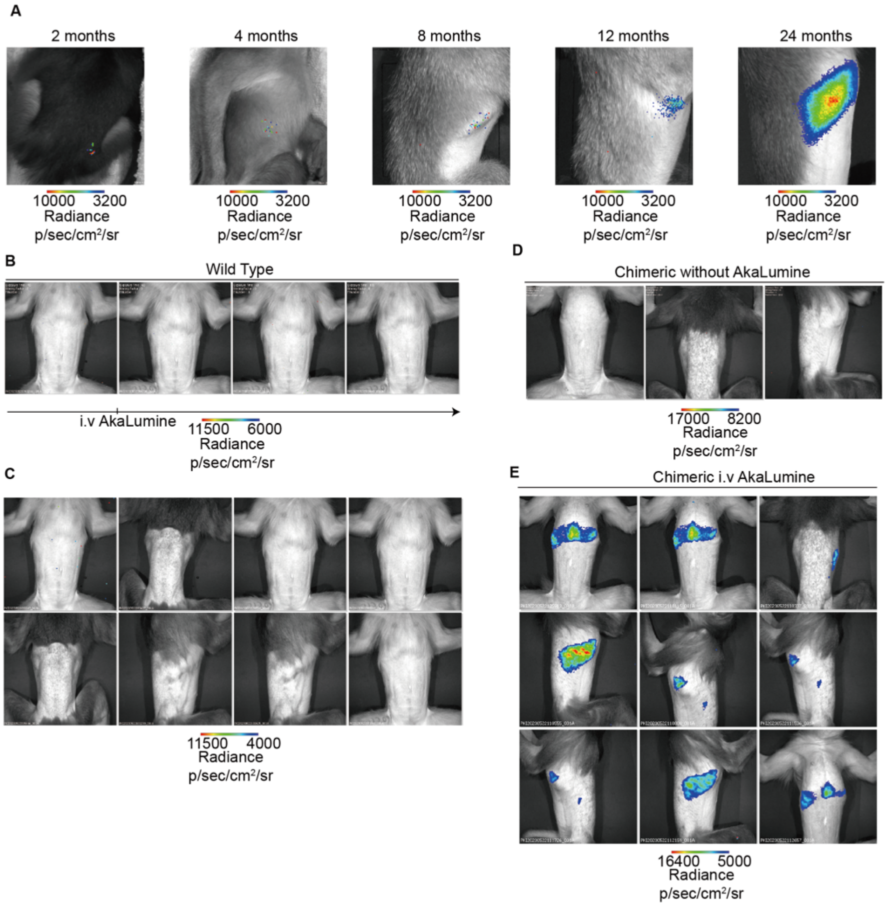

151

152

Figure S7

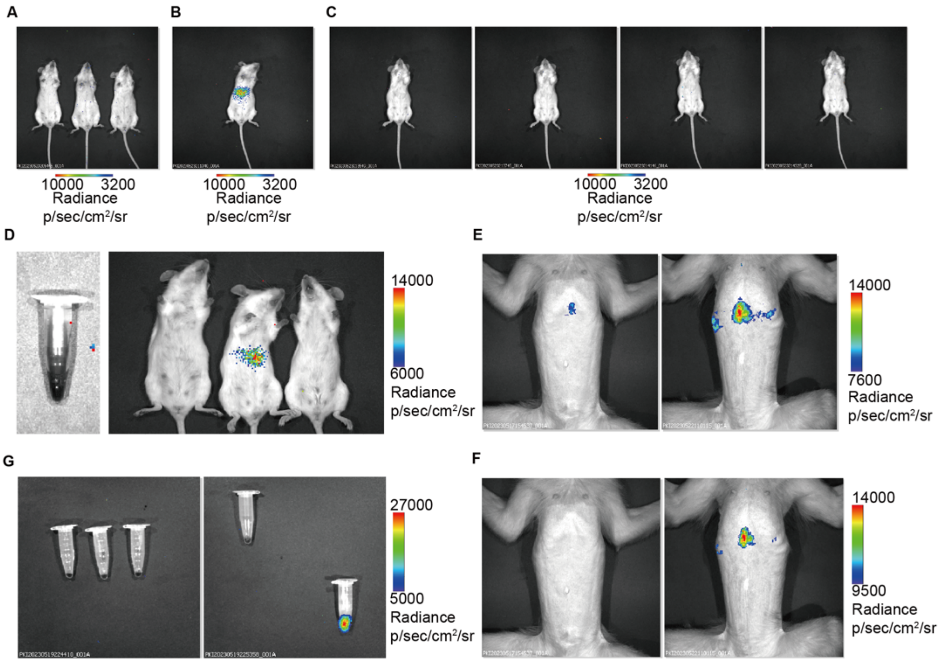

153
154

Figure S8

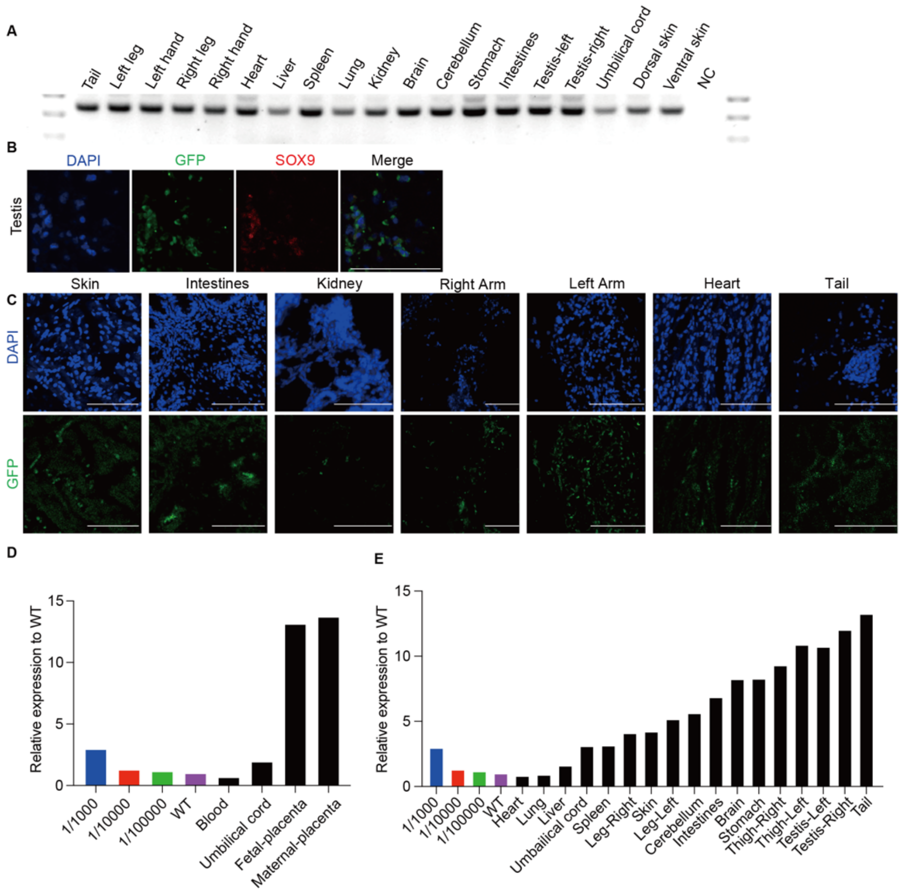

155
156

Figure S9

A

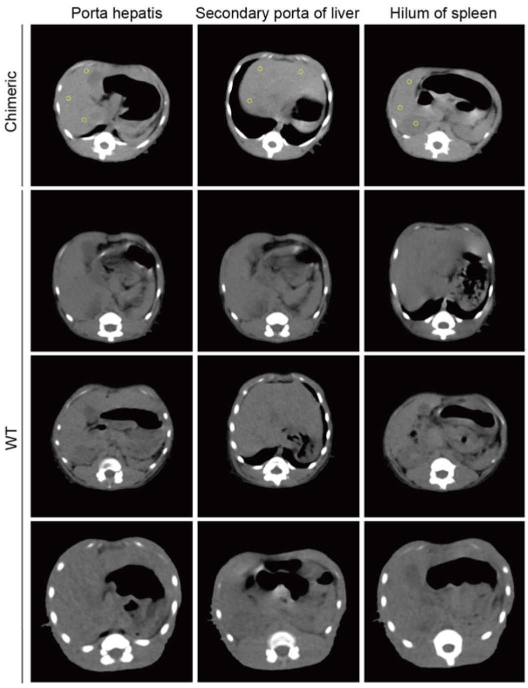
Porta hepatis
Secondary porta of liver
Hilum of spleen
Chimeric
WT

Figure S10

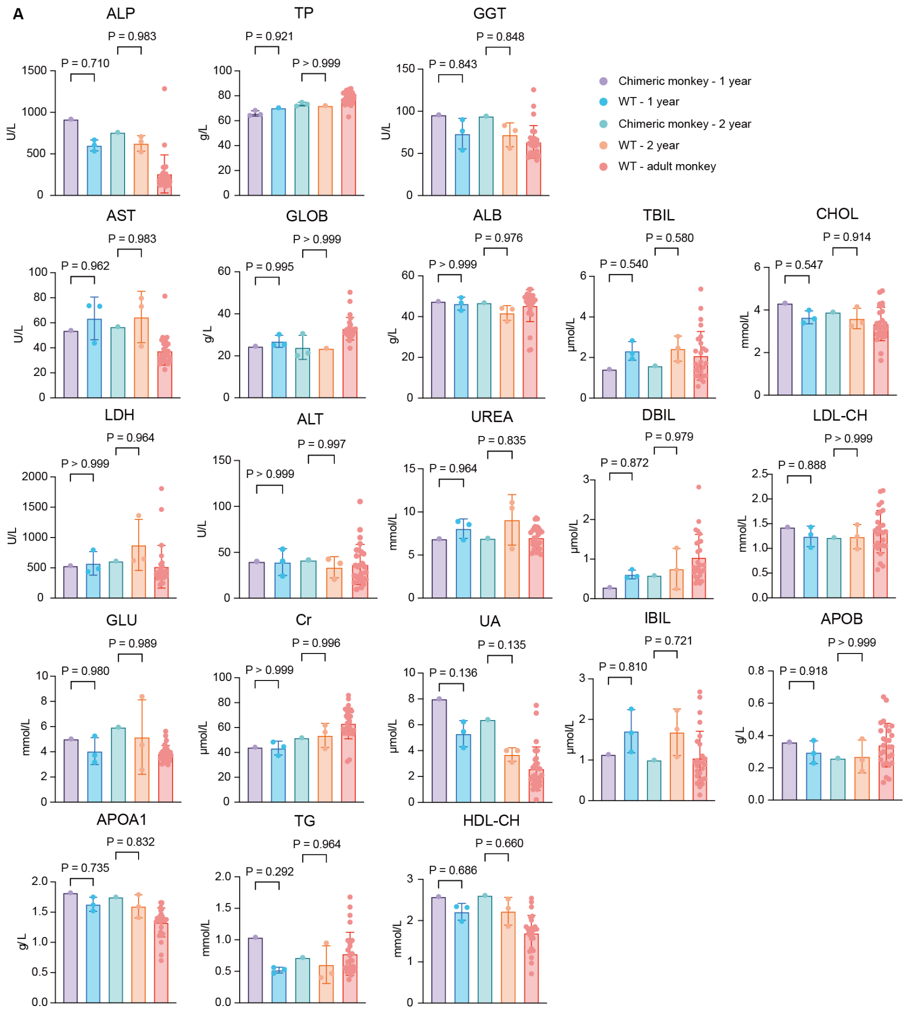
A
ALP
TP
GGT
AST
GLOB
ALB
TBIL
CHOL
LDH
ALT
UREA
DBIL
LDL-CH
GLU
Cr
UA
IBIL
APOB
APOA1
TG
HDL-CH
Chimeric monkey - 1 year
WT - 1 year
Chimeric monkey - 2 year
WT - 2 year
WT - adult monkey

Table S1. Summary of established monkey pluripotent stem cells

Cell lines	species	source	IF	EB	Teratoma	Karyotype	Single cell clone efficiency	Long term culture	Chimeric donor
CES1_1-XF PSC	monkey	primed CES1_1	yes	yes	yes	yes	-	>30 passages	-
CES_N	monkey	primed CES_N	yes	yes	-	-	-	>40 passages	chimeric monkey
XF-iPSC-142012	monkey	primed iPSC-142012	yes	-	-	-	-	>20 passages	-
XF-iPSC-182004	monkey	primed iPSC-142012	yes	yes	yes	-	-	>20 passages	-
XF-NTES-1	monkey	primed NTES-1	yes	yes	yes	yes	-	>20 passages	-
XF-NTES-2	monkey	primed NTES-2	yes	-	-	yes	-	>20 passages	-
XF-NTES-3	monkey	primed NTES-3	yes	-	-	yes	-	>10 passages	-
XF-ESC_1	monkey	blastocyst	yes	yes	yes	yes	yes	>30 passages	chimeric embryo culture
XF-ESC_2	monkey	blastocyst	yes	yes	yes	yes	yes	>30 passages	-
XF-ESC_3	monkey	blastocyst	yes	yes	yes	yes	yes	>30 passages	-
XF-iPSC	monkey	fibroblast	yes	yes	yes	yes	-	>20 passages	-

-: Not analyzed

Table S2. Conditions for the bioluminescence imaging experiments

	Sample	Emission filter	System	Field of view	f number	Binning	Exposure time	Figure
in vivo monkey	2 month monkey	open	IVIS Lumina LT	12.5 cm	1	8	60 sec	Figure 4e and S6a
	4 month monkey	open	IVIS Lumina LT	12.5 cm	1	8	60 sec	Figure 4e and S6a
	8 month monkey	open	IVIS Lumina LT	12.5 cm	1	8	60 sec	Figure 4e and S6a
	12 month monkey	open	IVIS Lumina LT	12.5 cm	1	8	180 sec	Figure 4e
	12 month monkey	open	IVIS Lumina LT	12.5 cm	1	8	60 sec	Figure S6a
	24 month monkey	open	IVIS Spectrum	13.2 cm	1	16	120 sec	Figure 4e and S6a
	wild-type monkeys	open	IVIS Lumina LT	12.5 cm	1	8	60 sec	Figure S4b
	wild-type monkeys	open	IVIS Spectrum	22.2 cm	1	8	60 sec	Figure S6b,c,S7e,f
	26 month monkey	open	IVIS Spectrum	22.2 cm	1	8	60 sec	Figure S6d,e,S7e,f
Cell	CMV-Akaluc-EF-1α-copGFP+ cells	open	IVIS Lumina LT	12.5 cm	1	8	Auto	Figure S4a
	no cell	open	IVIS Spectrum	13.2 cm	1	8	60 sec	Figure S4c
	CMV-Akaluc-EF-1α-copGFP+ cells	open	IVIS Spectrum	13.2 cm	1	8	Auto	Figure S4d
	no cell	open	IVIS Spectrum	13.2 cm	1	8	60 sec	Figure S7d
in vivo Mouse	Mouse	open	IVIS Spectrum	13.2 cm	1	8	60 sec	Figure S7a,b,c
	Mouse	open	IVIS Spectrum	13.2 cm	1	8	60 sec	Figure S4f
	Mouse	open	IVIS Lumina LT	12.5 cm	1	8	60 sec	Figure S4g
